# Oxidative Release of *O*-Glycans under Neutral Conditions for Analysis of Glycoconjugates Having Base-Sensitive Substituents

**DOI:** 10.1021/acs.analchem.3c00127

**Published:** 2023-06-01

**Authors:** Gaël M. Vos, Julia Weber, Igor R. Sweet, Kevin C. Hooijschuur, Javier Sastre Toraño, Geert-Jan Boons

**Affiliations:** † Department of Chemical Biology and Drug Discovery, Utrecht Institute for Pharmaceutical Sciences, Utrecht University, Universiteitsweg 99, 3584 CG Utrecht, The Netherlands; ‡ Complex Carbohydrate Research Center and Department of Chemistry, University of Georgia, 315 Riverbend Road, Athens, Georgia 30602, United States

## Abstract

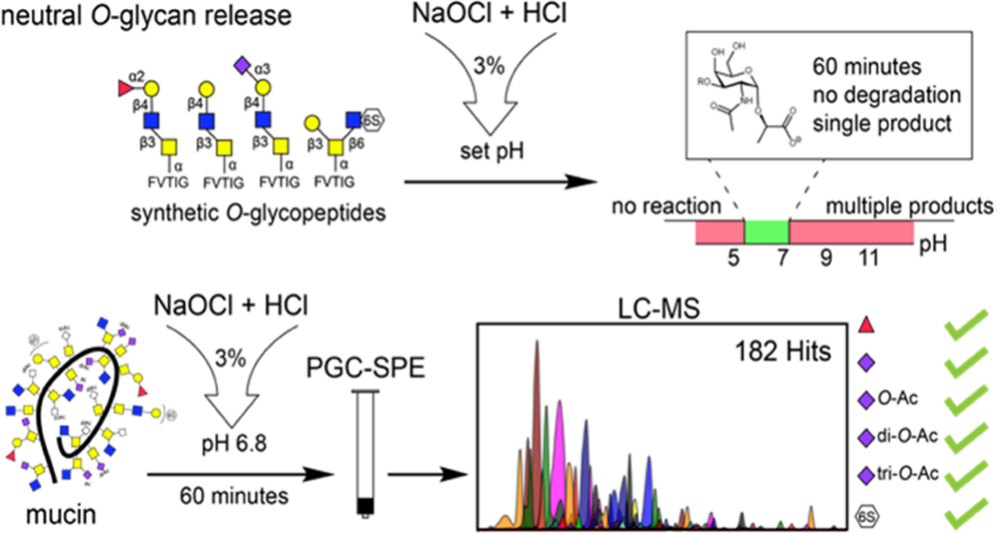

Protein *O*-glycosylation is one of the most diverse post-translational modifications. A critical step in the analysis of *O*-glycomes is the release of glycans from glycoconjugates. Current release methods rely mainly on β-elimination, which can result in peeling reactions and loss of base-sensitive functionalities leading to misidentification of glycans. To address this challenge, well-defined synthetic glycopeptides were used to establish a robust workflow for the oxidative release of *O*-glycans suitable for glycomics. Treatment of *O*-glycopeptides with neutralized hypochlorite resulted in the selective formation of lactic/glycolic acid glycosides, thereby retaining unique information of the parent amino acid (serine/threonine) that is lost by β-elimination. It locks the glycan in a closed ring configuration, thereby preventing peeling, and furthermore, the carboxylate of the anomeric tag promotes ionization in negative ion mode mass spectrometry, thereby increasing signal intensities. Labile modifications such as sialic acids, sulfates, and acetyl esters are maintained during the release procedure. The promise of the approach was demonstrated by the analysis of *O-*glycans from bovine submaxillary mucin, which identified mono- and di-*O-*acetylated sialoglycans as well as previously undetected tri-*O*-acetylated and sulfated glycans. The use of well-defined glycopeptide standards made it also possible to identify reaction intermediates, which in turn allowed us to postulate a reaction mechanism for oxidative *O*-glycan release under neutral conditions.

## Introduction

The most prominent post-translational modification of proteins in terms of complexity and diversity is by complex glycans.^1,2^ Almost all naturally occurring protein glycosylation can be classified as either *N*- and *O*-glycans by modification of side chains of Asn and Ser/Thr, respectively. The biosynthesis of *N*-linked glycans in vertebrates occurs by *en bloc* transfer of a dolichol-linked Glc_3_Man_9_GlcNAc_2_ to an Asn-X-Ser/Thr sequon of newly synthesized polypeptides, which is catalyzed by an oligosaccharide transferase complex.^3^ Subsequent trimming of the transferred oligosaccharide results in the formation of a Man_3_GlcNAc_2_ core structure that can be modified into complex structures. Mucin-type *O*-glycosylation is initiated by 20 polypeptide GalNAc-transferases that attach an α-linked *N*-acetyl-galactosamine (GalNAc) residue to specific Ser/Thr residues of proteins.^4,5^ The resulting GalNAc moieties are then elaborated into various core structures, which can be extended by type 1 and type 2 oligo-*N*-acetyl lactosamine moieties and capped by several forms of sialic acid and histo-blood group antigens. *O*-glycosylation can result in considerable structural diversity, which is regulated in a developmental, spatial, and temporal manner.^6^ Mucins and mucin-like proteins, which are densely modified by *O*-glycans, are important for tissue lubrication and form a physical barrier that can inhibit pathogen infection.^7,8^ They also mediate interactions between cells and their local environment. Changes in *O*-glycosylation are associated with many diseases such as cancer, inflammation, and enteric infections.^9−11^ Analysis of glycans of glycoconjugates in complex biological samples is important for linking specific structures to biological properties and for biomarker discovery.^12,13^ A critical step in glycomics is the release of glycans from glycoconjugates for analysis by mass spectrometry (MS).^14^*N-*glycans can be enzymatically released under mild conditions by peptide-*N*4-(*N*-acetyl-α-glucosaminyl) asparagine amidase (PNGase)^15^ or various endo-β-*N*-acetylglucosaminidases.^16^ No broad acting enzymes are available for the release of *O-*glycans and therefore these are commonly detached by base-mediated β-elimination to produce reducing glycans.^17,18^ A major problem of this approach is that the acyclic forms of the reducing glycans can undergo peeling reactions by the removal of the acidic C-2 proton, resulting in the elimination of C-3-linked substituents.^19−21^ Chemical modifications have been explored to reduce peeling by, for example, reduction by sodium borohydride to give alditols that are resistant to peeling after conversion.^22,23^ Reductive amination can achieve a similar result while allowing the incorporation of chemical entities that may facilitate purification and/or ionization.^24−26^ These methods, however, still suffer from peeling and loss of base labile substituents such as acetyl esters. A mild *O-*glycan release method that does not result in peeling and is compatible with labile substituents is needed for the analysis of *O-*glycomes.

**Figure 1:**
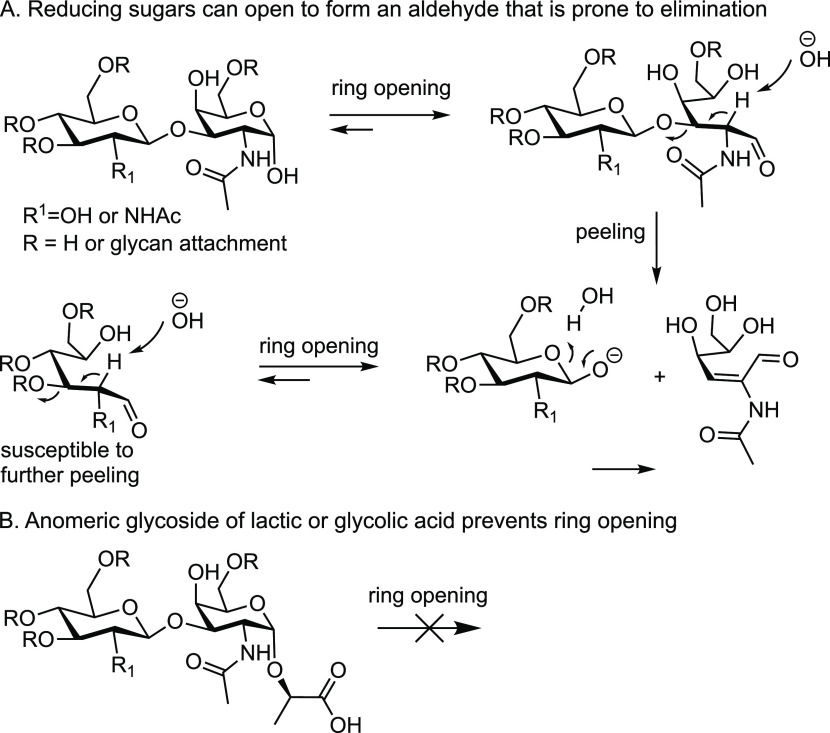
(A) *O*-glycans released by β-elimination are susceptible to peeling. In the case of reducing sugars (aldoses), there is an equilibrium between the open and closed ring configuration. The C-2 proton of the acyclic form (α to aldehyde) is acidic, which can cause β-elimination of C-3 linked substituents. A new reducing glycan is formed that can undergo further peeling reactions. (B) *O*-glycans obtained by oxidative release have an anomeric glycolic/lactic acid moiety (glycoside) that prevents ring opening. As a result, the aldehyde containing acyclic form cannot be formed. In the cyclic form, the C-2 proton is not acidic, which prevents degradation of the glycan structure.

Alkaline hypochlorite is an inexpensive and scalable method for oxidative release of *N-*glycans, *O-*glycans, and glycolipids.^27^ Under these oxidative conditions, glycolipids are converted into glycan nitriles while *N*-glycans react to free reducing structures. In the case of *O-*glycans, it produces several products, including glycosides of glycolic or lactic acid and reducing sugars, which are prone to peeling.^27−29^ Here, we employed well-defined synthetic glycopeptides to establish a robust workflow for the oxidative release of *O-*glycans suitable for glycomic analysis, preserving sensitive substituents. Surprisingly, it was found that *O*-glycans can be released by neutralized hypochlorite, which results in the selective formation of lactic/glycolic acid-linked *O-*glycan, thereby giving unique information of the parent amino acid (serine/threonine) that is lost by β-elimination. It locks the glycan in a closed ring configuration, thereby preventing peeling reactions (Figure 1). An additional advantage of the procedure is that the anomeric tag contains a carboxylate that promotes ionization in negative ion mode MS, thereby increasing signal intensities and improving limits of detection. It also alters the fragmentation pattern of released *O*-glycans during multistage MS analysis with respect to the analysis of free reducing end or reduced *O*-glycans. Moreover, labile modifications such as sialic acid, sulfates, and acetyl esters are maintained during the release procedure. The promise of the approach was demonstrated by the analysis of *O-*glycans from bovine submaxillary mucin (BSM), which identified mono*-* and di-*O-*acetylated sialoglycans as well as previously undetected tri-*O*-acetylated and sulfated glycans. The use of well-defined glycopeptide standards made it also possible to identify reaction intermediates, which in turn made it possible to postulate a reaction mechanism for oxidative *O*-glycan release under neutral conditions.

## Experimental Section

### Materials

Bovine submaxillary mucin (BSM), trifluoroacetic acid (TFA), hydrogen chloride (HCl), sodium borohydride, ammonium bicarbonate, acetic acid, and formic acid were obtained from Sigma-Aldrich (Saint Louis, MO). Acetonitrile (ACN; LC-MS grade) was purchased from Biosolve B.V. (Valkenswaard, The Netherlands). Hypercarb Hypersep porous graphitized carbon solid-phase extraction (PGC-SPE) cartridges with a bed weight of 25 mg and NaOCl (10–15% active chlorine) were acquired from Thermo Fisher Scientific (Waltham, MA). Ultrapure water was produced by a Synergy UV water purification system from Merck Millipore (Burlington, MA).

### Release of *O-*Glycan from Glycopeptides **1–4**

The pH of a 15% NaOCl solution was adjusted to the corresponding pH (5–12) by titrating 5 mL of the hypochlorite solution with 1 M HCl and adjusting the volume to 8 mL with water. A volume of 25 μL of NaOCl of this solution was added to 50 μL of glycopeptide solution in water (1 mg/mL), which was kept on ice to minimize potential glycan degradation. At 10 min intervals, a 5 μL volume of the mixture was transferred to a new Eppendorf tube, and the reaction was quenched with 3 μL 1% formic acid. The mixture was freeze-dried, redissolved in 25 μL of water, purified by PGC-SPE, and analyzed by liquid chromatography (LC)-MS.

### Release of *O-*Glycan from BSM

For oxidative release, the pH of a 5 mL 15% NaOCl solution was adjusted to 6.8 by titration with approx. 3 mL of 1 M HCl. A volume of 100 μL of this solution was added to a 200 μL BSM solution in water (1 mg/mL), which was kept on ice to minimize potential degradation. At 30 min intervals, a sample of 50 μL of the reaction mixture was taken, and the reaction was quenched with 3 μL of 10% (v/v) formic acid. The mixture was freeze-dried, redissolved in 25 μL water, subjected to PGC-SPE, and analyzed by LC-MS. For release by β-elimination, 1 mg of BSM was dissolved in 400 μL of 55 mg/mL sodium borohydride solution in 0.10 M NaOH. The reaction mixture was incubated at 50 °C for 16 h and then quenched by dropwise addition of glacial acetic acid to a final pH of 5.^30,31^ The quenched mixture was diluted to 1 mL with water, subjected to PGC-SPE, and analyzed by LC-MS.

### PGC-SPE Purification of Released Glycans

SPE cartridges were equilibrated with water. Aqueous samples were loaded onto the cartridge, the cartridge was washed with 1 mL of water, and glycans were eluted with 1 mL of ACN:0.1% TFA 60:40% (v/v). The solvents were evaporated under a stream of nitrogen and reconstituted for (LC)-MS analysis.

### LC-MS Analysis

LC-MS was performed on an Agilent Technologies (Santa Clara, CA) Infinity 1290 LC system coupled via a dual-source AJS electrospray interface to an Agilent Technologies 6560B ESI Ion Mobility Q-TOF. Glycopeptide standards were analyzed with a SeQuant ZIC-HILIC column (20 × 2.1 mm^2^, 3.5 μm particles; Merck, Darmstadt, Germany), with 0.1% (v/v) formic acid as eluent A and ACN as eluent B, using a linear gradient from 90–50% B over 5 min and maintaining 50% B for 8 min. MS was performed in positive ion mode for glycans **1** and **3** and negative ion mode for glycans **2** and **4**. *O-*glycans, released from BSM oxidatively or by β-elimination, were separated with a ZIC-HILIC column (150 × 2.1 mm^2^, 3 μm particles) with a ZIC-HILIC guard column (20 × 2.1 mm^2^, 3 μm particles; Merck, Darmstadt, Germany) using 5 mM ammonium formate (pH 6.5) as eluent A and ACN as eluent B. Chromatographic separation was achieved using 85% B for 5 min, followed by a linear gradient to 50% B over 25 min at 0.2 mL/min. *O*-glycans released by β-elimination were additionally analyzed with the same gradient using 10 mM ammonium bicarbonate (pH 7.8) as eluent A to preserve sulfated moieties better. MS analysis was performed in negative ion mode with a capillary voltage of 3500 V, nozzle voltage of 2000 V, nebulizer pressure of 40 psi, drying gas flow rate of 300 °C at 8 L/min, and sheath gas temperature of 300 °C at 11 L/min.

## Results and Discussion

### Development of the Oxidative Release Workflow

Recent developments in the synthesis of *O-*glycopeptides provide access to well-defined *O-*glycopeptide standards.^32,33^ We anticipated that such standards would be useful to establish workflows for the controlled release of *O*-glycans. Furthermore, it was expected that well-defined glycopeptides can also be useful to detect intermediate products, which, in turn, can give insights into the reaction mechanism of glycan release.

**Figure 2:**
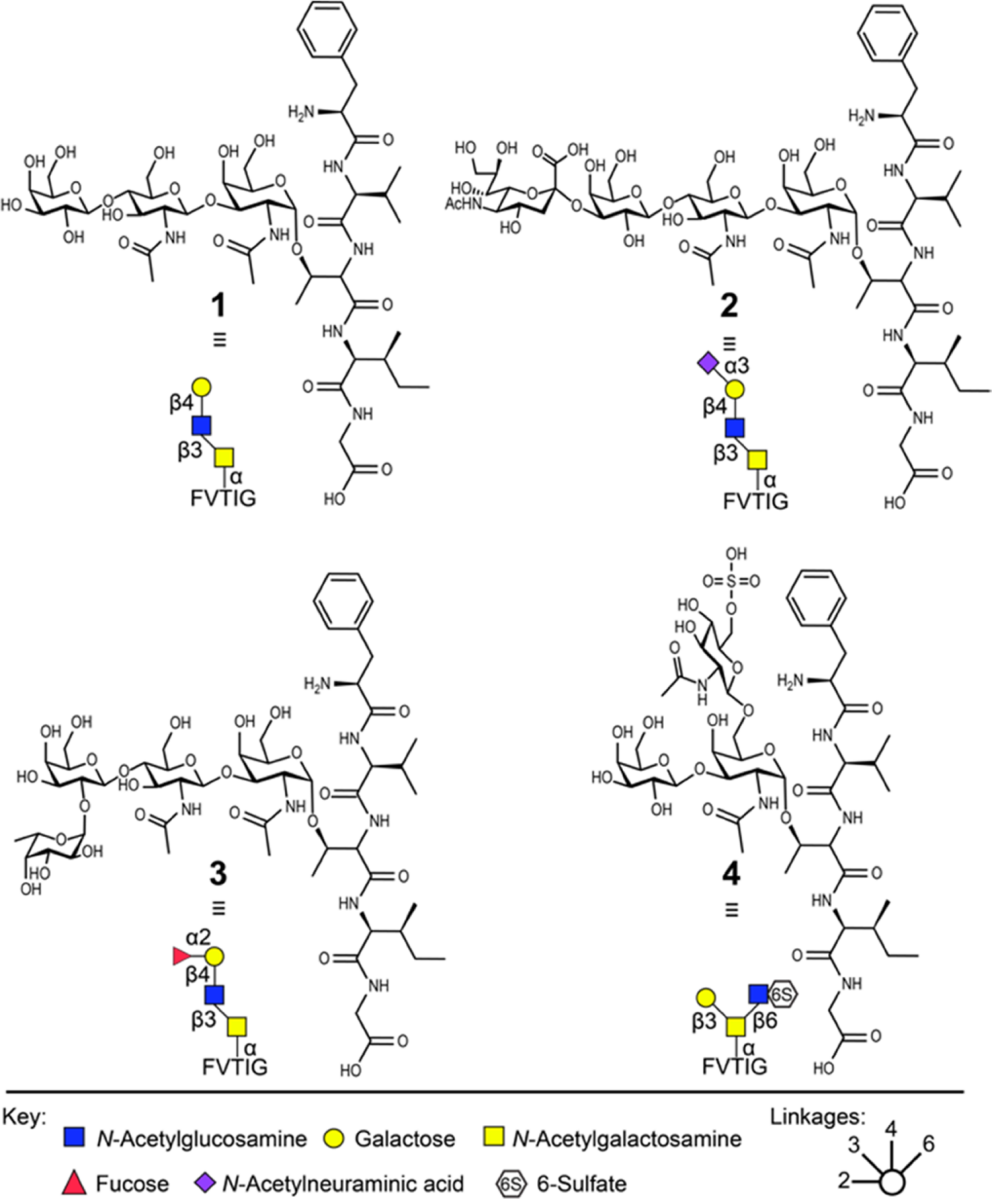
Chemical structures and graphic representation of the *O*-glycopeptide standards with FVTIG peptide sequence.

To develop an oxidative release workflow suitable for *O*-glycomic analysis, glycopeptides **1**–**4** were prepared. [Weber et al.; manuscript in preparation] All compounds contain a 3-*O*-substituted GalNAc moiety, making them prone to peeling (Figure 2). Compounds **1**–**3** are core-3 glycopeptides, including neutral glycan (**1**) and structures decorated with sialic acid (**2**) and fucose (**3**). Compound **4** is a core-2 glycopeptide having a sulfate at the C-6 position of GlcNAc. Glycopeptide **1** was used as an initial standard to investigate *O-*glycan release by hypochlorite. Glycopeptides **2**–**4** were then used to determine the chemical stability of these common glycan substructures under the optimized release conditions. First, the effect of pH of hypochlorite on reaction time and product formation was investigated using core-3 glycopeptide **1**.

**Figure 3:**
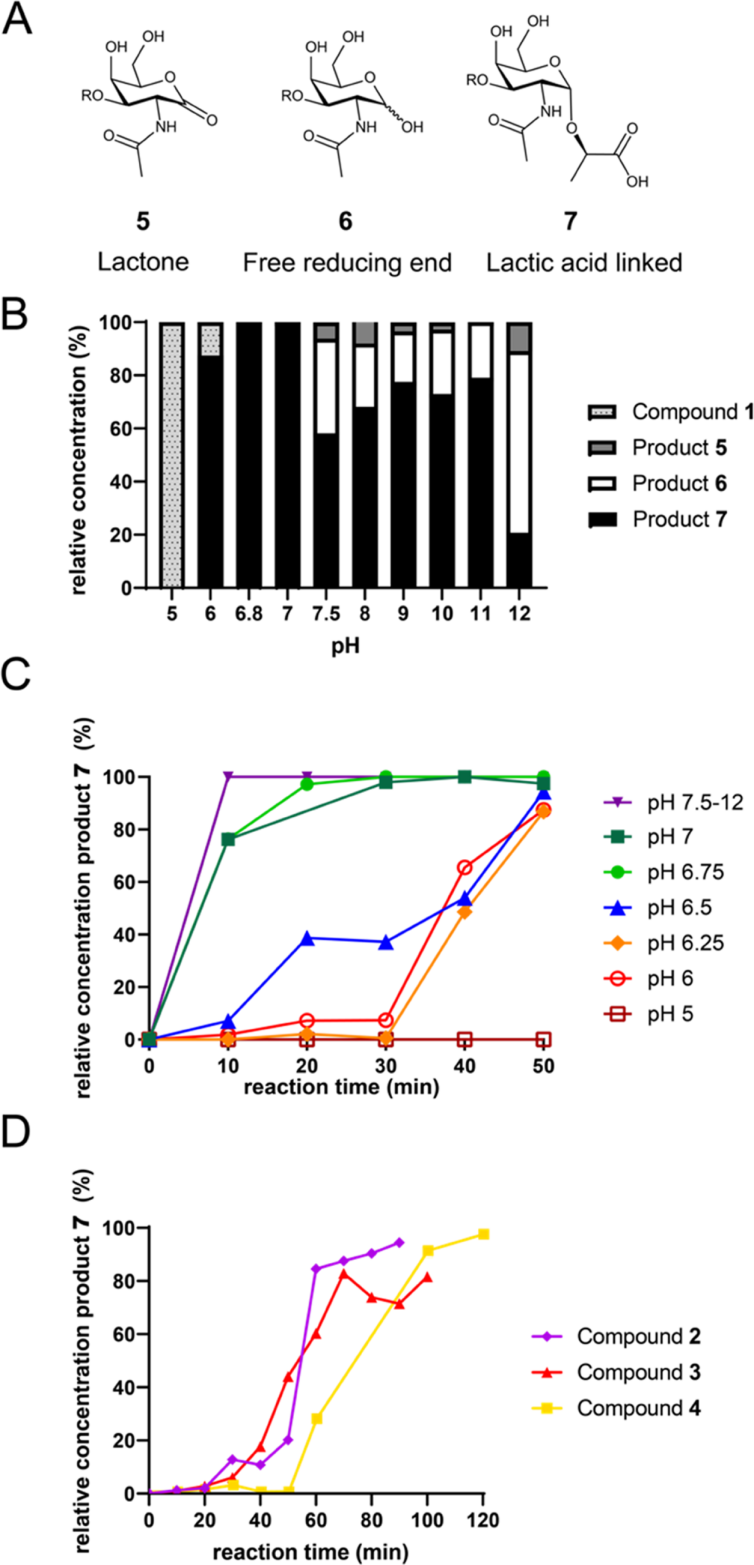
(A) Chemical structure of products generated by oxidative release. (B) Relative concentrations by MS of *O*-glycopeptide standard 1 and reaction products after 60 min of incubation in a 3% hypochlorite solution at 0 °C and different pH values. (C) Relative concentrations by MS of released amounts of 1 over time in a 3% hypochlorite solution at 0 °C, measured as lactic acid-linked glycoside **7** signal relative to starting material and observed side product signals. (D) Relative concentrations by MS of released amounts of **2–4** over time in a 3% hypochlorite solution at 0 °C and pH 6.8, measured as lactic acid-linked glycoside **7** signals relative to starting material and observed side product signals.

Thus, this compound was subjected to a 3% sodium hypochlorite solution, which was acidified with 1 M HCl, covering a pH range from pH 5 to pH 12, keeping the final hypochlorite concentration constant. The reactions were performed in an ice bath to minimize potential degradation by β-elimination of the reactions performed under alkaline conditions. Samples were taken at 10 min time intervals, reactions were quenched with formic acid, and samples were subjected to solid-phase extraction (SPE) using porous graphitized carbon cartridges before analysis by LC-MS. Surprisingly, complete consumption of the starting material was observed within 10 min in the pH range 7.5–12, which is much shorter than previously reported.^27−29^ The major release product under the most alkaline condition (pH 12) was free reducing carbohydrate 6 (approx. 68% as relative concentration determined by MS). Both lactone **5** and lactic acid-linked *O-*glycan **7** were also observed (Figure 3A–C). In the pH range 7.5–11, the major product of the reaction was lactic acid-linked *O-*glycan, but the lactone-containing product and free reducing glycan were also formed, albeit at lower relative quantities compared to treatment at pH 12. Further acidification to pH 7–5.5 resulted in the clean formation of lactic acid-linked products. The reaction at neutral pH did result in a somewhat slower *O-*glycan release. At a pH below 5.5, no reaction was observed, and only unreacted glycopeptide was detected. Neutralized hypochlorite in a pH range of 6.8–7 resulted in complete *O-*glycan release within 30 min, with no detectable side reactions and therefore all further experiments were conducted within this pH range.

Sialic acid, fucose, and sulfate containing glycopeptides **2**, **3**, and **4** were subjected to hypochlorite neutralized to pH 6.8 to evaluate the stability of common glycan epitopes. The reactions were sampled in 10 min intervals, and reaction progress and glycan stabilities were evaluated by LC-MS. All evaluated glycan epitopes were converted to the lactic acid glycosides within 60–90 min and remained stable under the neutralized hypochlorite conditions (Figure 3D). Only minimal Neu5Ac hydrolysis was observed, which might be attributed to the acidic quenching conditions. Lactic acid-linked glycoside was the only release product observed for glycopeptide **4** and accounted for 93% of the relative abundance observed for glycopeptide **2** and 97% for glycopeptide **3**; the remainder was free reducing carbohydrate. Notably, a delay in the consumption of starting material was observed for all substituted glycopeptides. This delay can possibly be explained by the buildup of Cl_3_O_2_^–^, which is a more reactive chlorinating agent suggested to be formed in neutralized hypochlorite.^34^ Treatment during 60 min with 3% hypochlorite at pH 6.8 and 0 °C resulted in complete conversion of glycopeptides **1**–**3** to the lactic acid-linked *O-*glycan, while sulfated glycopeptide **4** required a treatment time of 90 min for complete release.

### Oxidative Release of *O*-Glycans from Submaxillary Mucin

**Figure 4:**
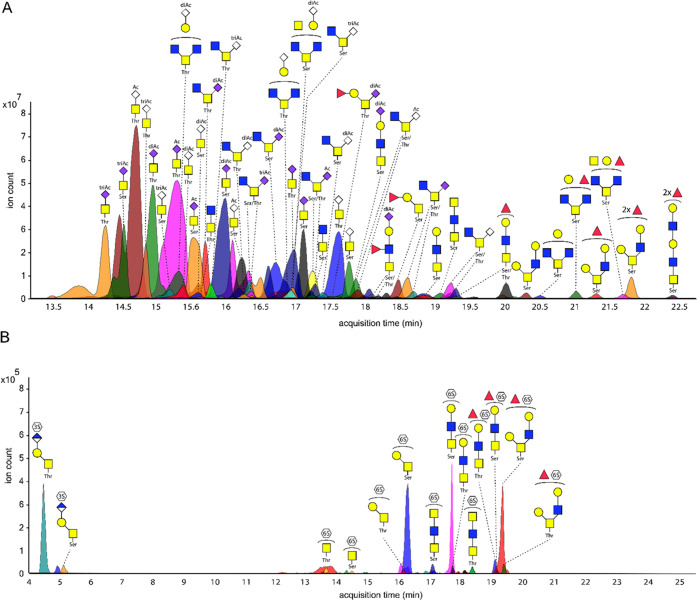
(A) Suggested structures of *O*-glycans from bovine submaxillary mucin (BSM) with >0.1% relative concentration released using neutralized hypochlorite at pH 6.8 for 60 min. (B) Suggested structures of sulfated *O*-glycans from bovine submaxillary mucin (BSM) released using neutralized hypochlorite at pH 6.8 for 60 min.

Bovine submaxillary mucin (BSM) was subjected to the optimized oxidative release conditions. BSM is a commercially available mucin that is relatively well characterized and commonly employed in biomedical research.^27,35−37^ It carries sialoglycans modified by acetyl esters^38^ that are prone to migration and hydrolysis under alkaline conditions.^39^ Thus, BSM was treated with neutralized hypochlorite, and samples were taken at 30 min intervals to confirm the previous established release kinetics. Comparable results were obtained at 30 and 60 min intervals for smaller glycans, but larger glycans were detected at higher relative abundances after 60 min treatment. Surprisingly, prolonged exposure to the hypochlorite solution (90 and 120 min) resulted in the formation of previously undetected chlorinated products. Therefore, a 60 min release time was selected at which minimal chlorination was observed (<0.5% for the most abundant *O-*glycan). It resulted in the identification of 275 *O-*glycans, which were either modified by an anomeric lactic or glycolic acid derived from threonine or serine, respectively (Figure 4A). Chromatographic separation of isomeric *O*-glycan cores 3 and 5 by ZIC-HILIC was not achieved and therefore putative *O*-glycan structures are reported, based on the most common *O*-glycan cores found in BSM.^35^ Gratifyingly, no reducing glycans or other derivatives were observed in notable quantities. Furthermore, most of the abundant *O*-glycans were detected both as lactic and glycolic acid-linked products, thereby confirming the *O*-glycan structure.

The released glycans from BSM consisted mostly of sialylated di- and trisaccharides but also included larger compounds such as a low abundant dodecasaccharide. The majority (92% of the total ion abundance) of all detected structures contained sialic acids, with Neu5Ac and Neu5Gc accounting for 73 and 27% of the sialic acid content, respectively. Fucose was detected on 56 *O-*glycans, accounting for 3.8% of the total glycan abundance. Eight di-fucosylated structures were observed (0.7% of the total glycan abundance), indicating the presence of Lewis^y^ or Lewis^b^ epitopes. Furthermore, 36 structures, accounting for 1% of the total glycan abundance, contained both a fucoside and sialosides, probably representing sialyl Lewis^x^ or sialyl Lewis^a^ epitopes.

The presence of acetyl ester and sulfated *O-*glycans in BSM was evaluated to confirm that neutralized hypochlorite is suitable to release such saccharides. Acetyl esters were observed on 84 *O-*glycans as mono-, di-, and tri- *O-*acetylated sialic acids. The presence of acetyl esters was detected on 87% of all Neu5Ac and 74% of all Neu5Gc-containing ions, supporting high preservation of this functionality. Furthermore, sialic acids having multiple acetyl esters were detected in substantial quantities (Neu5Ac: mono 38%; di 37%; tri 12%) (Neu5Gc: mono 33%; di 25%; tri: 16%). The presence of these di- and tri-*O-*acetylated sialic acids shows that even the very labile acetyl esters on the C-7 and C-8 positions are preserved under neutralized conditions.

A total of 14 sulfated structures were detected, accounting for 0.4% of the total ion abundance, which to the best of our knowledge, is the first time that such structures have been detected on BSM (Figure 4B). Fucosylated and sulfated structures were observed, but none of these compounds contained sialic acid, which is in stark contrast to the highly sialylated unsulfated glycans. Surprisingly, two HNK-1 epitope-containing *O-*glycans were detected, composed of a core 1 trisaccharide. The conservation of labile di- and tri-*O-*acetylated sialic acids and sulfated *O-*glycans released from BSM supports the attractiveness of the release method for the analysis of complex mucinous samples. In this respect, the previous analysis only provided mono-acetylated derivatives.^27^

BSM was also subjected to reductive β-elimination under standard conditions, which gave 40 different structures using an LC eluent at pH 6.5 (Table III, Supporting Information) and 57 structures using an LC eluent pH of 7.8 (Table IV, Supporting Information). Predominantly, *O*-glycans with negatively chargeable moieties such as sulfates and sialosides were detected. As expected, no acetylated structures were found after β-elimination due to the requirement of a high pH. The fact that the oxidative release method gave a substantially larger number of structures (Table II, Supporting Information) can be attributed to preservation of base labile glycans, better ionization of neutral *O*-glycans that are equipped with a chargeable anomeric moiety, and discrimination of threonine- and serine-linked *O*-glycans. It is important to note that the treatment with neutralized sodium hypochlorite and β-elimination provide different structures (lactic and glycolic acid glycosides vs alditols), and thus, in MS analysis, different ions are observed, which may also exhibit different ionizability and fragmentation properties.

### Reaction Mechanism for Oxidative Release of *O*-Glycans from Glycopeptides and Glycoproteins

**Figure 5:**
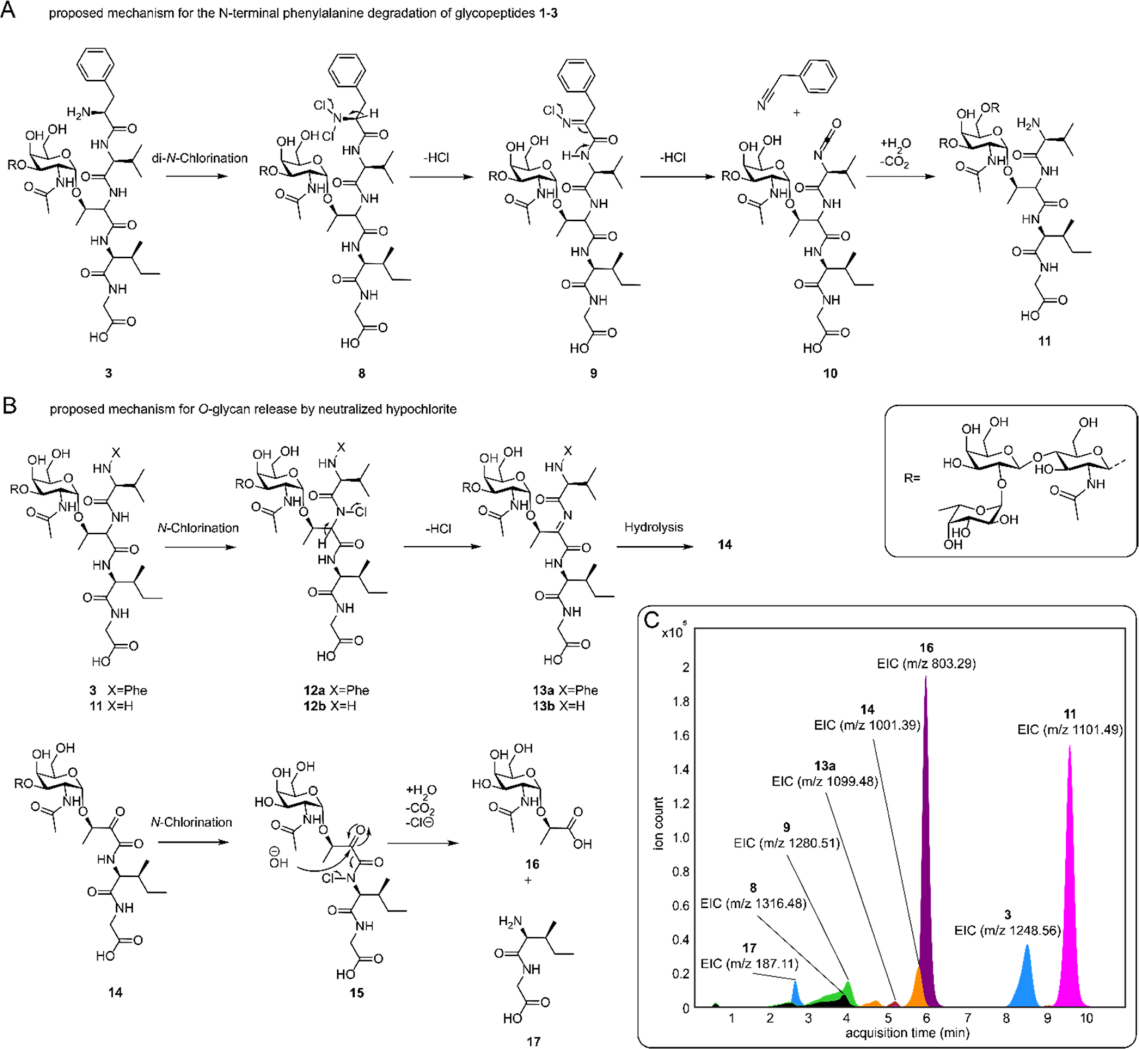
(A) Proposed mechanism for the *N*-terminal phenylalanine degradation of glycopeptides **1**–**3** mediated by hypochlorite. (B) Proposed reaction mechanism for the formation of lactic acid-linked *O*-glycan by hypochlorite-mediated *O*-glycan release. (C) Extracted ion chromatograms of compound **3** with proposed intermediates and reaction products after 50 min of incubation with neutralized hypochlorite at pH 6.8 obtained by LC-MS in negative ion mode.

The synthetic glycopeptide standards made it possible to identify reaction intermediates of the oxidative release reactions. The identified intermediates were consistently observed for glycopeptides **1**–**4** when released at neutral pH and made it possible to postulate a reaction mechanism (Figure 5). Intermediate **11** (*m*/*z* 1101.49) was abundantly detected and consistent with a loss of phenylalanine. The masses of **8** (*m*/*z* 1316.48) and **9** (*m*/*z* 1280.51) correspond to the di-chlorinated starting material and mono-chlorinated imine, which is most like a result of elimination. Detection of **13a**, which has an *m*/*z* −2 with respect to compound **11** (*m*/*z* 1099.48), indicates the formation of an imine derivative of **11**. Intermediate **14** (*m*/*z* 1001.39) was observed in relatively low abundances for all standards, corresponding to a loss of the complete *N*-terminal peptide with respect to the glycosylation site and formation of an α-ketoamide.

*N-*terminal degradation to give **11** is probably initiated by *N-*terminal *N-*dichlorination to give intermediate **8**, which can be converted into *N-*chloroiminopeptide **9** by an elimination reaction. Subsequent hydrolysis of **9** and decarboxylation results in the formation of **11** and 2-phenylacetonitrile.^40^ Surprisingly, compound **4** did not show substantial *N-*terminal degradation while showing similar release times as glycopeptides **1**–**3**. Further *N-*terminal degradation of valine following this mechanism would generate a cyanomethyl glycoside, which was not observed. Although hydrolysis of the cyanomethyl glycoside could produce **16**, we excluded this degradation pathway since these cyanomethyl glycosides have been reported as stable products produced by the alkaline oxidative release of glycolipids.^27^ Therefore, we propose that hypochlorite-mediated *O*-glycan release is not dependent on *N*-terminal degradation, and a critical step is chlorination of the backbone amide between GalNAc-Thr and valine (Figure 5B) to give compound **12**. This reaction can occur on starting material **3** as well as on partially degraded products such as intermediate **11** to give **12a** and **12b**, respectively. Compounds **12a,b** can eliminate to give imines **13a,b**, which can hydrolyze, resulting in the formation of α-ketoamide **14**. Hydrolysis of **14** takes place at the more nucleophilic ketone moiety and leads to the lactic acid **16**. Detection of dipeptide **17** supports this pathway and indicates a decarboxylative process. *N*-Chlorination of the GalNAc/GlcNAc amide and related degradation products were not observed at the investigated time points. Chlorination of amides occurs through corresponding iminols,^41^ and the propensity of peptide bonds to tautomerize may provide a rationale for the selective degradation of the peptide backbone.^42^ The electron-withdrawing carbohydrate moiety may influence tautomerization and chlorination, and further studies are required to investigate possible preferred peptide bonds for chlorination.

## Conclusions

An oxidative release method for *O*-glycans is described. The method uses neutralized hypochlorite, which preserves labile entities such as acetyl esters. It provides glycosides of lactic and glycolic acid, which prevents peeling and increases the sensitivity of detection by MS. The presence of the latter tag also provides a convenient filtering approach to avoid false positive hits, which is expected to be especially useful in the *O*-glycomic analysis of complex biological samples. The use of well-defined glycopeptides made it possible to examine in detail the product formation and reaction kinetics of the oxidative release of *O*-glycans, and it was found that the formation of glycosides of lactic and glycolic acid was surprisingly fast. A core-2 glycopeptide having a sulfate at the C-6 position of GlcNAc reacted slower than the other glycopeptides. However, at a 60 min time point, substantial glycan release was observed while minimizing byproduct formation by chlorination. Treatment of BSM with neutralized sodium hypochlorite for 60 min did result in the release of glycan moieties such as that of **4**, and thus the method can detect such compounds. It is expected that the oxidative release protocol can be combined with the analysis of *N*-glycans. In this respect, *N*-glycans can be selectively cleaved by treatment with PNGase and retrieved for analysis, which can be followed by oxidative *O*-glycan release and further analysis. Such a protocol also removes possible interference of *N*-glycans for *O*-glycan release. We anticipate that neutralized hypochlorite *O*-glycan release will find broad use as a mild and efficient *O*-glycan release method that better reflects the native glycome.

## Supporting Information Available

Extracted ion chromatograms of glycopeptide standards **1–4**; *O*-glycan structures released with hypochlorite as well as *O*-glycan structures released from BSM with neutralized hypochlorite and by reductive β-elimination (PDF)
