# Supplementary material for: Oxidative Release of O-Glycans under Neutral Conditions for Analysis of Glycoconjugates Having Base-Sensitive Substituents
Source: Anal Chem. 2023 Jun 1;95(23):8825–33. doi: 10.1021/acs.analchem.3c00127 (PMC10267892; doi:10.1021/acs.analchem.3c00127)
Supplement: Supplementary file 1 — ac3c00127_si_001.pdf [file ac3c00127_si_001.pdf]

Supporting Information

Oxidative release of *O*-glycans under neutral conditions for analysis of glycoconjugates having base sensitive substituents

Gael M. Vos[1], Julia Weber[1], Igor R. Sweet[1], Kevin C. Hooijschuur[1], Javier Sastre Toraño[1], Geert-Jan Boons[1,2]

[1] Department of Chemical Biology and Drug Discovery, Utrecht Institute for Pharmaceutical Sciences, Utrecht University, Universiteitsweg 99, 3584 CG Utrecht, the Netherlands

[2] Complex Carbohydrate Research Center and Department of Chemistry, University of Georgia, 315 Riverbend Road, Athens, GA 30602, United States

Table of Content

Page S2-S20 **Table I.** Extracted LC-MS chromatograms of glycopeptide standards **1**-**4** and compounds released from the glycopeptide with hypochlorite.

Page S21-S31 **Table II.** Compositions of *O*-glycans released from BSM with neutralized hypochlorite and analyzed with LC-MS with an eluent pH of 6.5.

Page S32-S34 **Table III.** Compositions of *O*-glycans released from BSM with reductive beta elimination and analyzed with LC-MS with an eluent pH of 6.5.

Page S35-S36 **Table IV:** Compositions of *O*-glycans released from BSM with reductive beta elimination and analyzed with LC-MS with an eluent pH of 7.8.

Table I: Extracted LC-MS chromatograms of glycopeptide standards **1-4** and compounds released from the glycopeptide with hypochlorite. The lactic acid-linked glycoside is annotated as “released”.

Chromatograms of peptide reaction mixtures at different time points and pH values during oxidative release.

Glycopeptide	pH of hypochlorite solution	Reaction time (min)	Extracted ion chromatogram
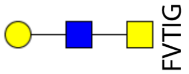	5.0	0 min	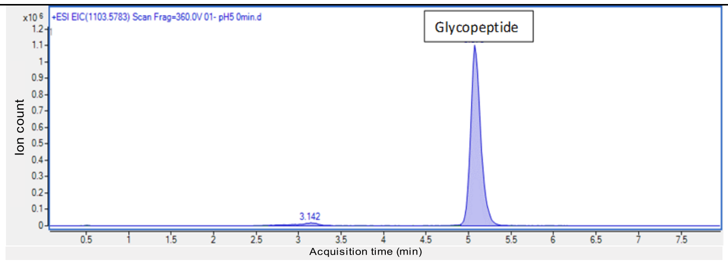
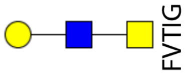	5.0	10 min	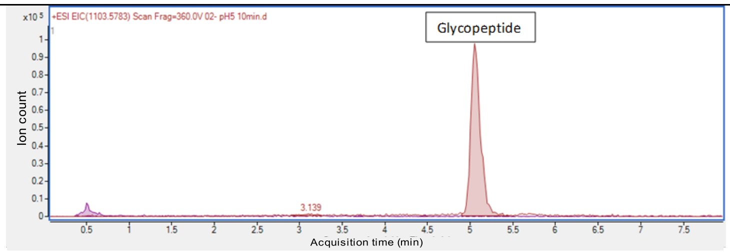
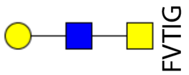	5.0	20 min	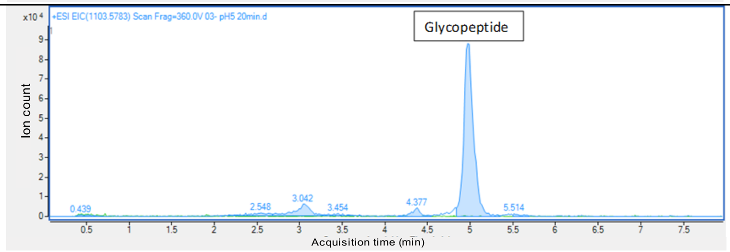
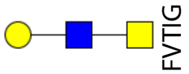	5.0	30min	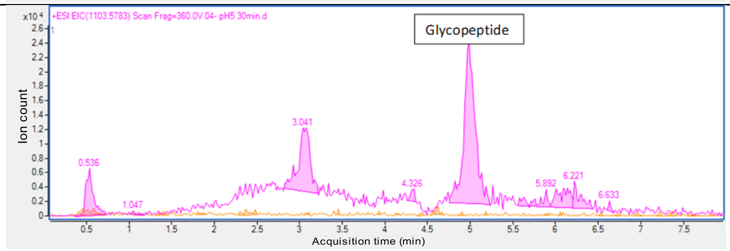
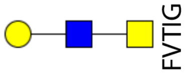	5.0	40 min	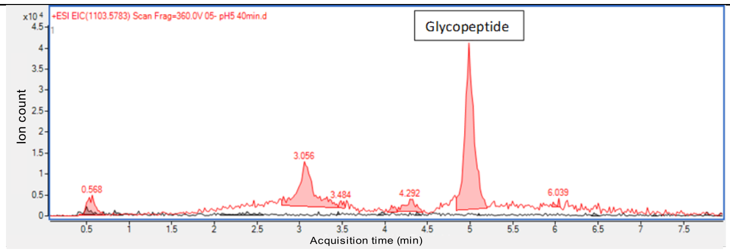

Chromatograms of peptide reaction mixtures at different time points and pH values during oxidative release.

Glycopeptide	pH of hypochlorite solution	Reaction time (min)	Extracted ion chromatogram
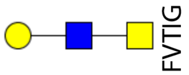	5.0	50 min	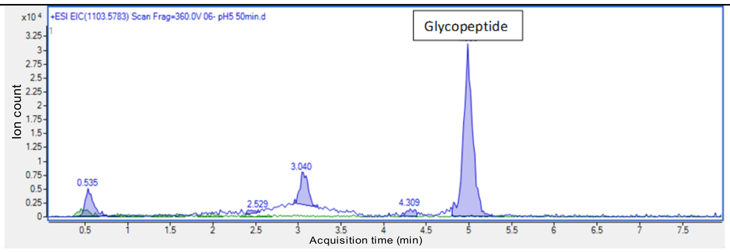
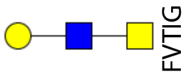	5.0	60 min	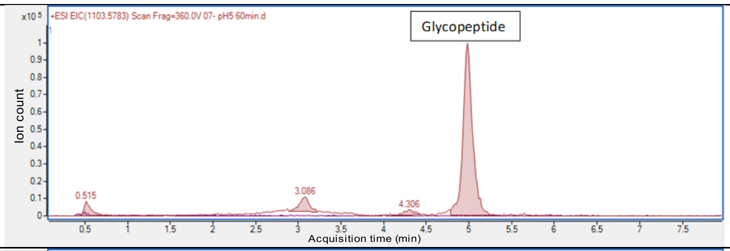
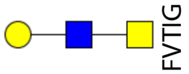	6.0	0 min	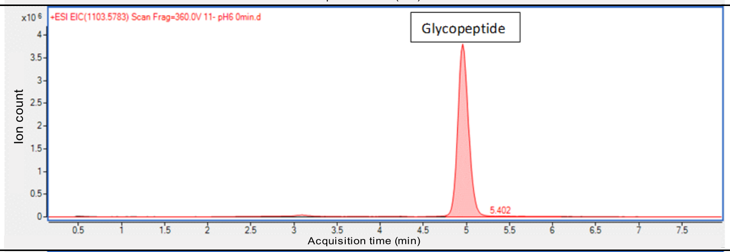
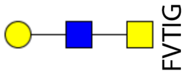	6.0	10 min	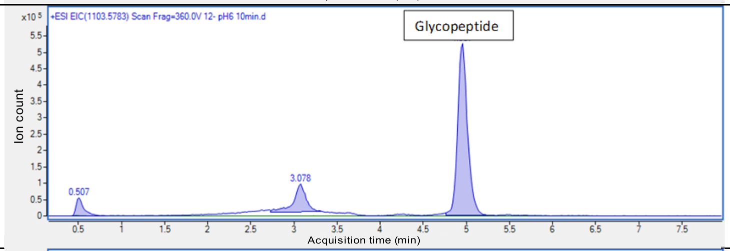
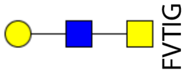	6.0	20 min	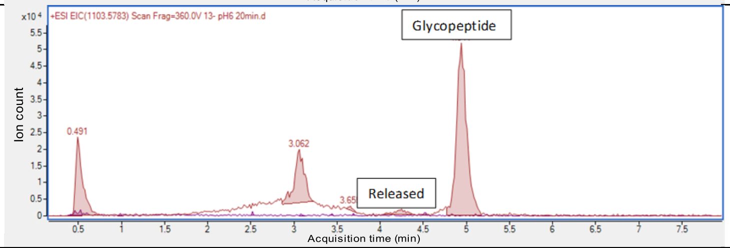
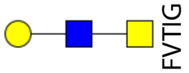	6.0	30min	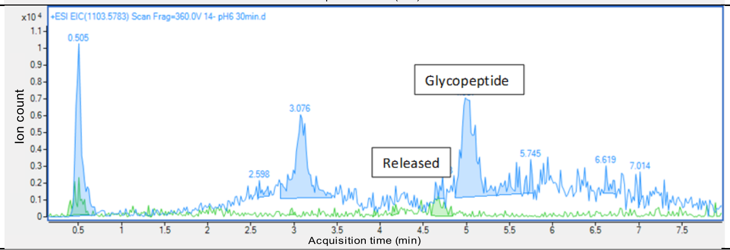

Chromatograms of peptide reaction mixtures at different time points and pH values during oxidative release.

Glycopeptide	pH of hypochlorite solution	Reaction time (min)	Extracted ion chromatogram
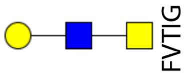	6.0	40 min	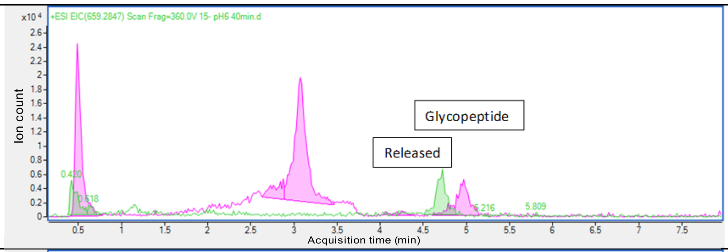
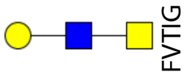	6.0	50 min	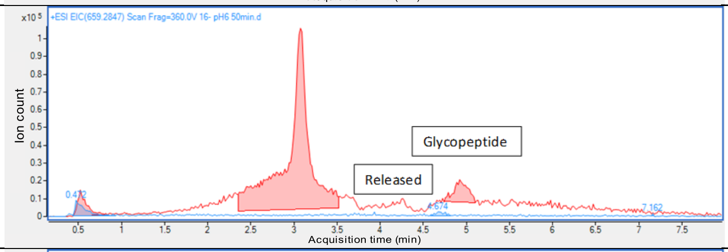
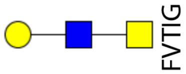	6.0	60 min	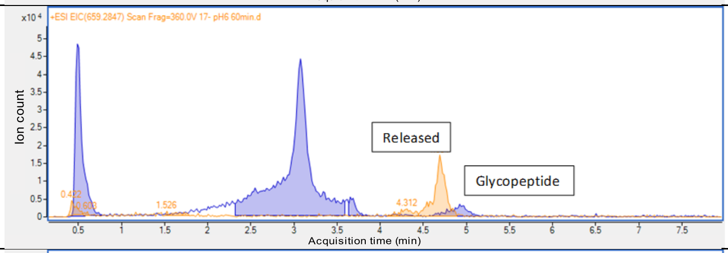
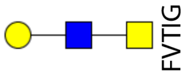	6.25	0 min	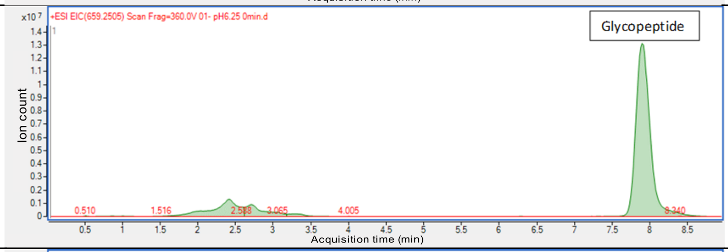
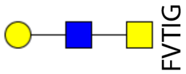	6.25	10 min	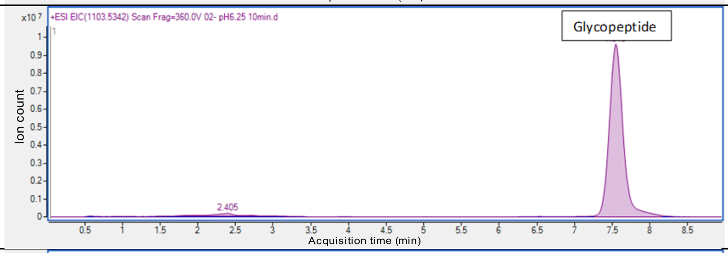
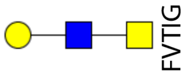	6.25	20 min	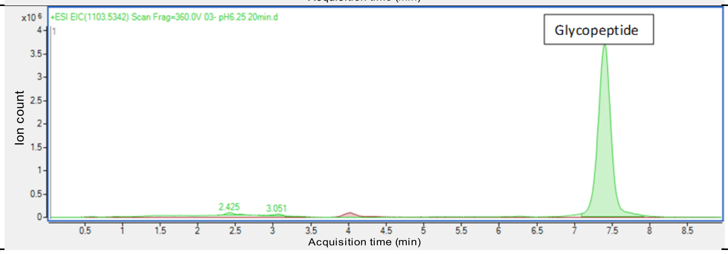

Chromatograms of peptide reaction mixtures at different time points and pH values during oxidative release.

<table>
<tr><th>Glycopeptide</th><th>pH of hypochlorite solution</th><th>Reaction time (min)</th><th>Extracted ion chromatogram</th></tr>
<tr><td>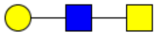 FVTIG</td><td>6.25</td><td>30min</td><td>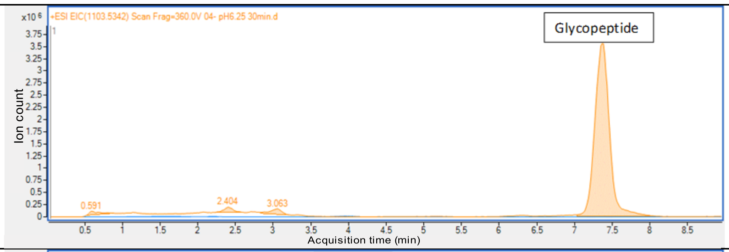
</td></tr>
<tr><td>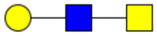 FVTIG</td><td>6.25</td><td>40 min</td><td>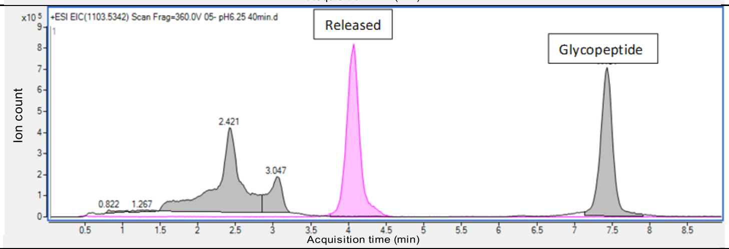
</td></tr>
<tr><td>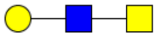 FVTIG</td><td>6.25</td><td>50 min</td><td>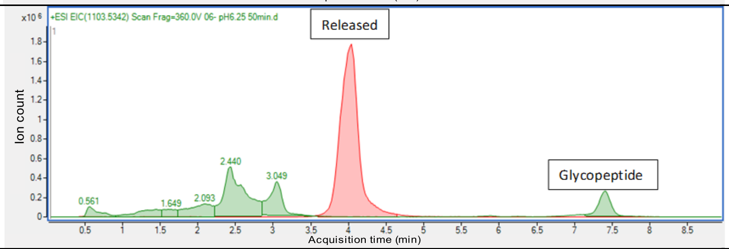
</td></tr>
<tr><td>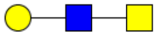 FVTIG</td><td>6.5</td><td>0 min</td><td>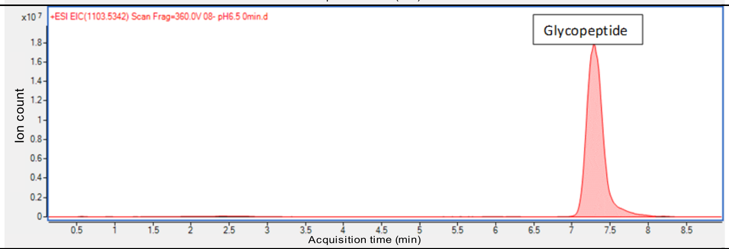
</td></tr>
<tr><td>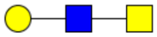 FVTIG</td><td>6.5</td><td>10 min</td><td>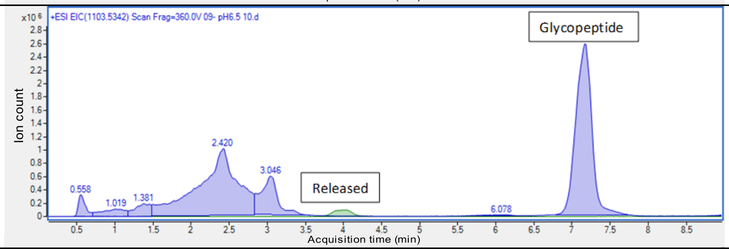
</td></tr>
<tr><td>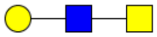 FVTIG</td><td>6.5</td><td>20 min</td><td>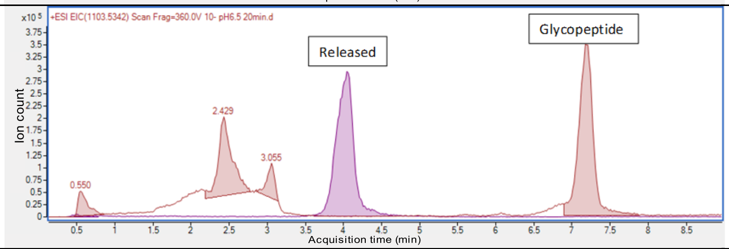
</td></tr>
</table>

Chromatograms of peptide reaction mixtures at different time points and pH values during oxidative release.

Glycopeptide	pH of hypochlorite solution	Reaction time (min)	Extracted ion chromatogram
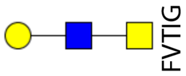	6.5	30min	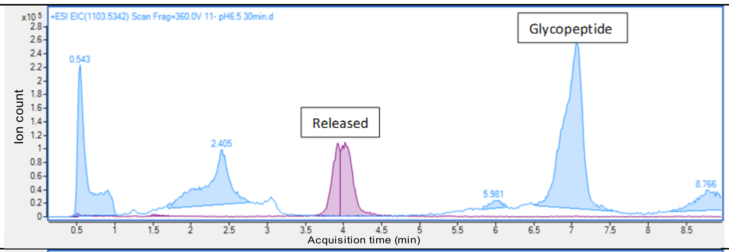
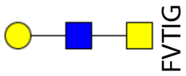	6.5	40 min	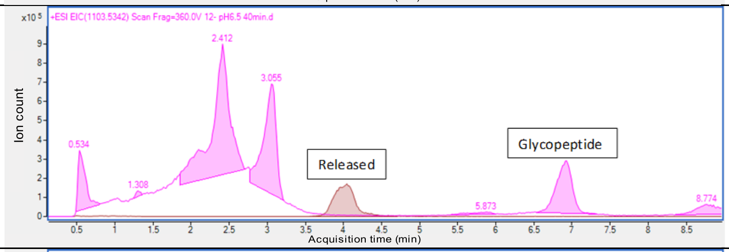
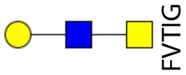	6.5	50 min	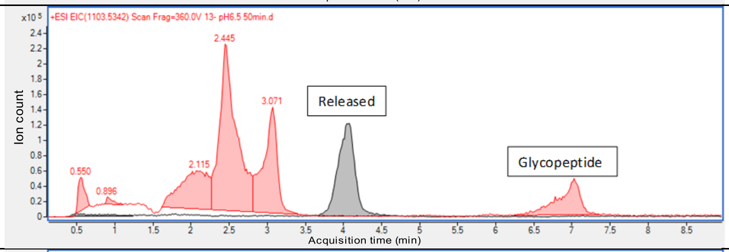
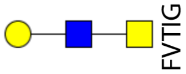	6.75	0 min	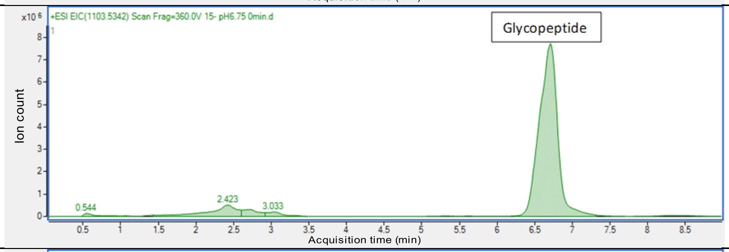
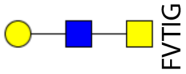	6.75	10 min	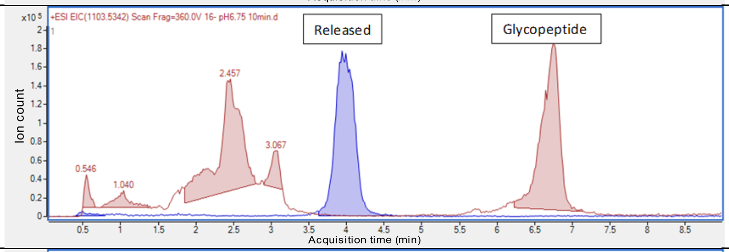
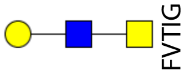	6.75	20 min	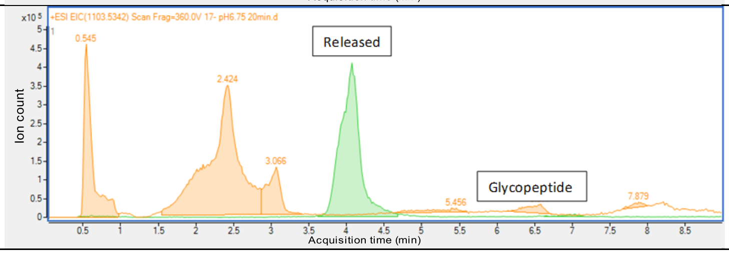

Chromatograms of peptide reaction mixtures at different time points and pH values during oxidative release.

Glycopeptide	pH of hypochlorite solution	Reaction time (min)	Extracted ion chromatogram
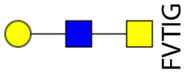	6.75	30min	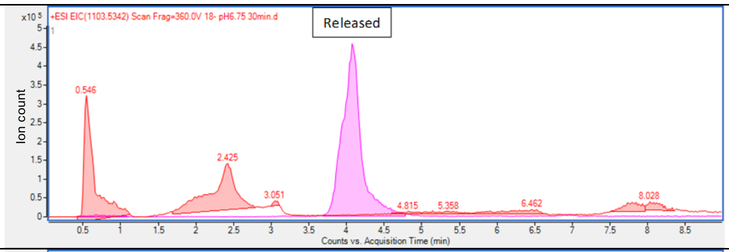
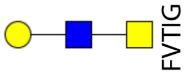	6.75	40 min	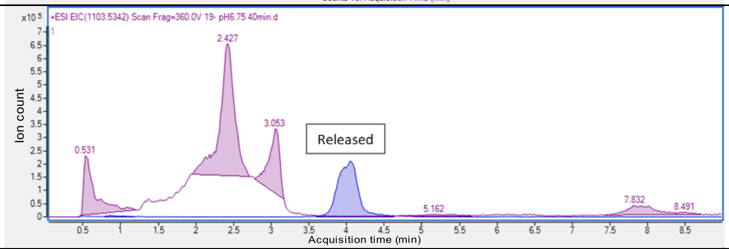
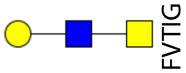	6.75	50 min	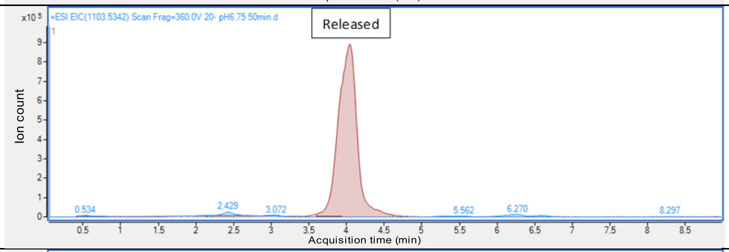
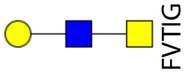	6.75	60 min	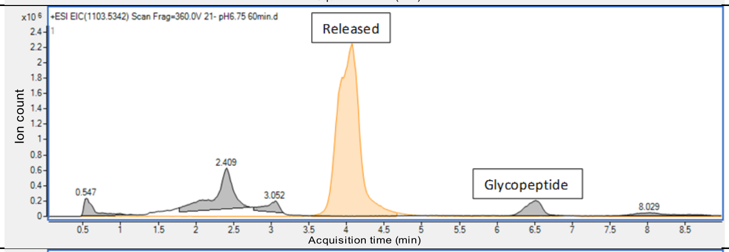
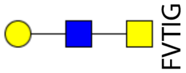	7.0	0 min	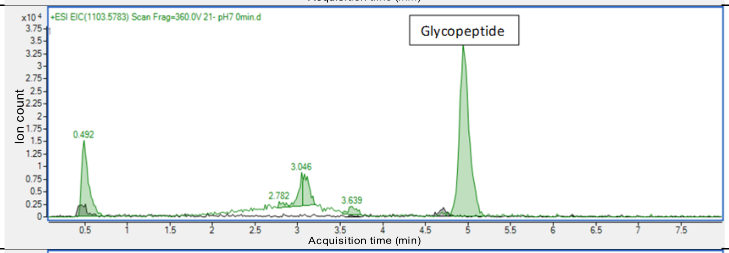
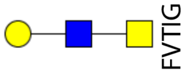	7.0	10 min	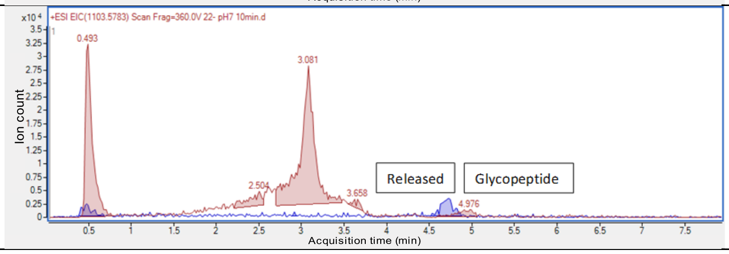

Chromatograms of peptide reaction mixtures at different time points and pH values during oxidative release.

Glycopeptide	pH of hypochlorite solution	Reaction time (min)	Extracted ion chromatogram
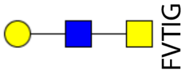	7.0	30min	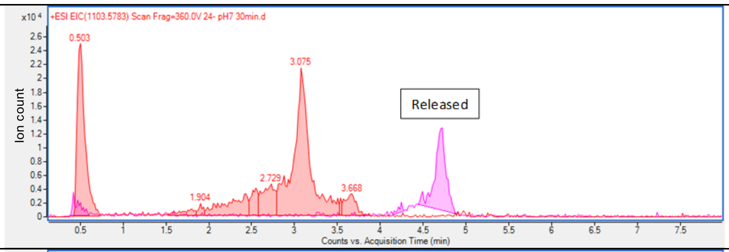
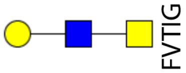	7.0	40 min	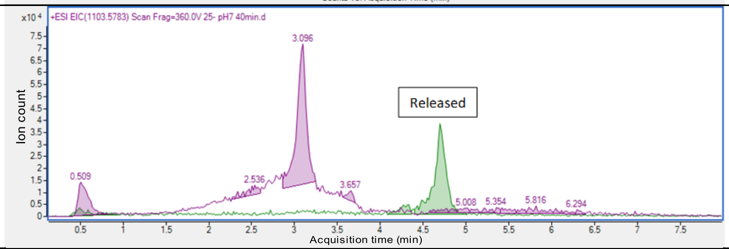
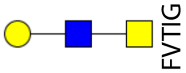	7.0	50 min	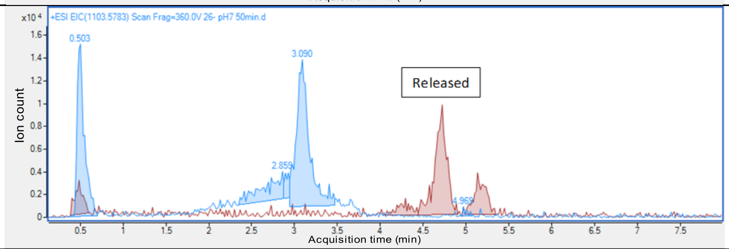
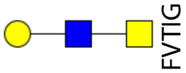	7.0	60 min	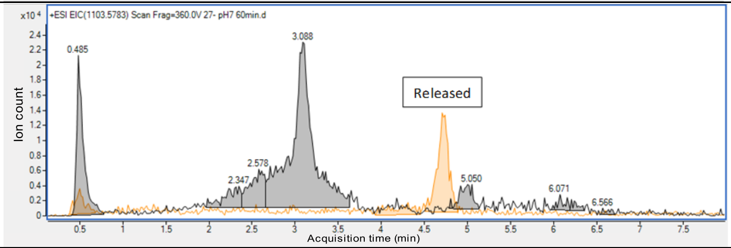
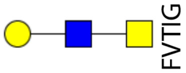	7.5	0 min	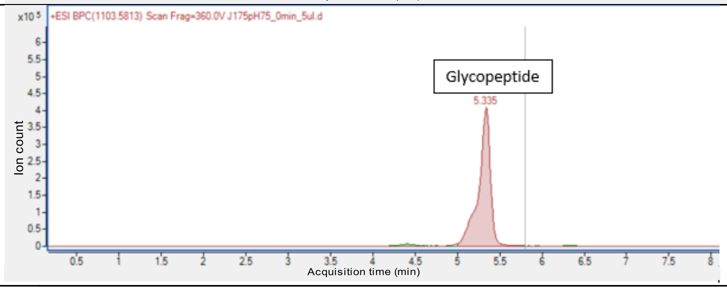
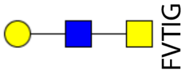	7.5	10 min	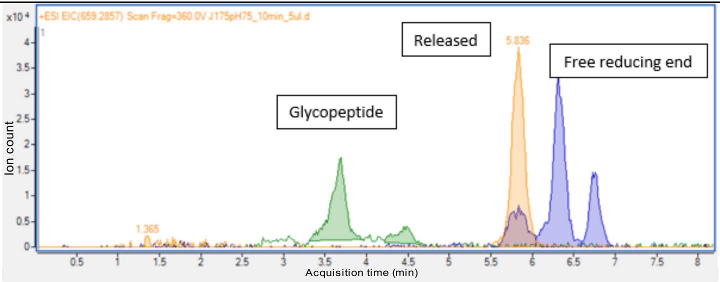

Chromatograms of peptide reaction mixtures at different time points and pH values during oxidative release.

Glycopeptide	pH of hypochlorite solution	Reaction time (min)	Extracted ion chromatogram
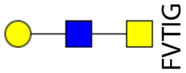	7.5	20 min	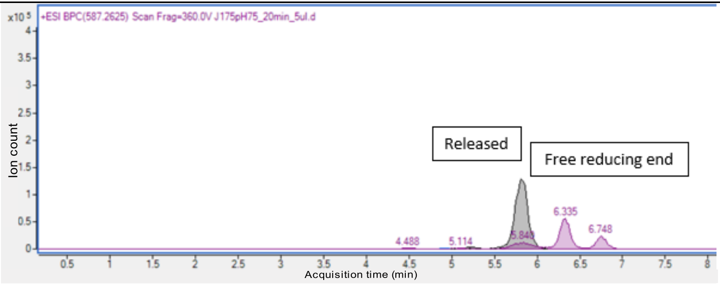
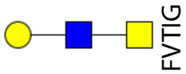	7.5	30min	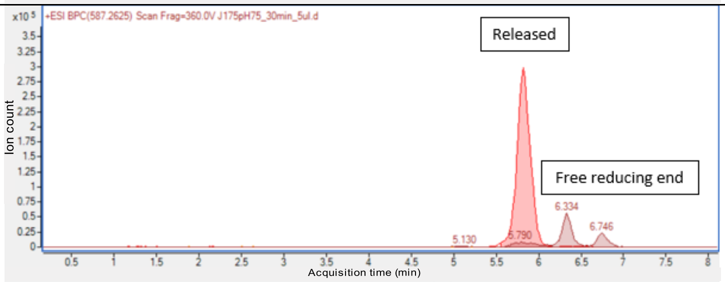
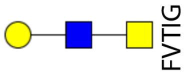	7.5	40 min	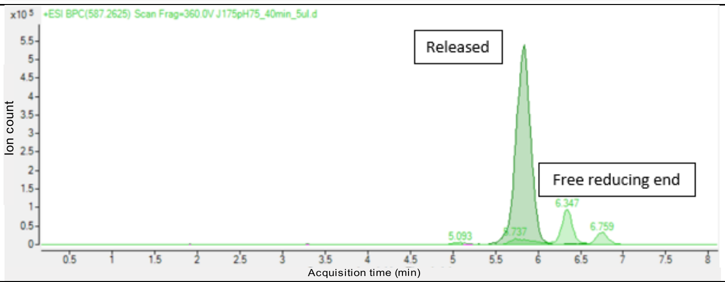
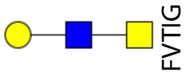	7.5	50 min	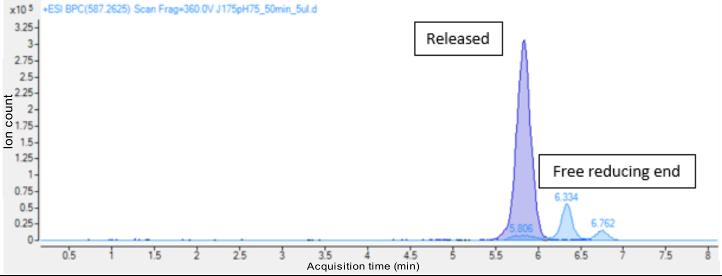
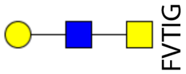	7.5	60 min	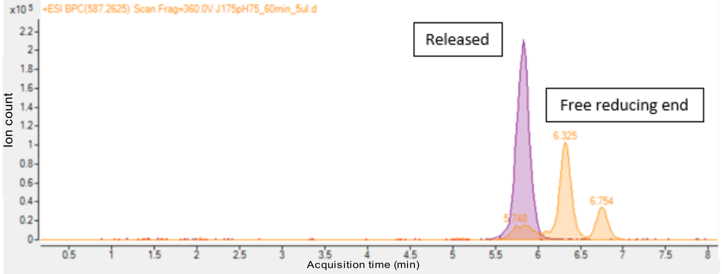

Chromatograms of peptide reaction mixtures at different time points and pH values during oxidative release.

Glycopeptide	pH of hypochlorite solution	Reaction time (min)	Extracted ion chromatogram
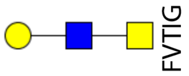	8	0 min	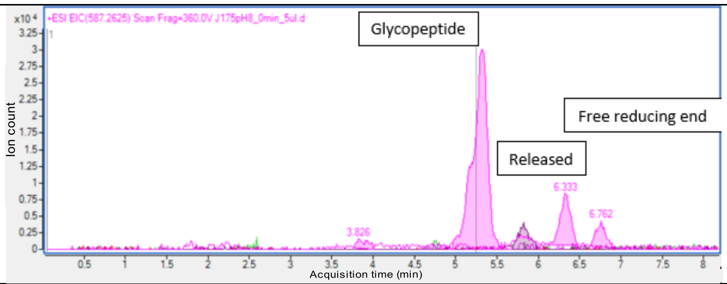
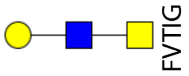	8	10 min	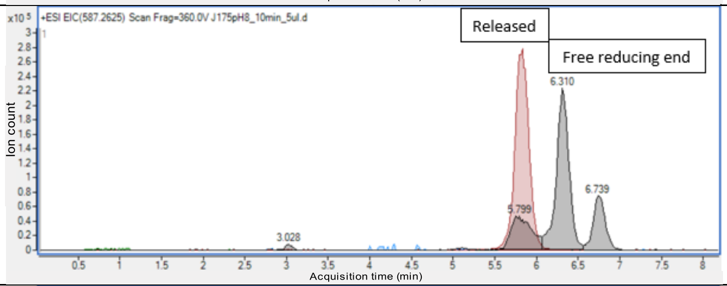
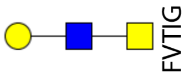	8	20 min	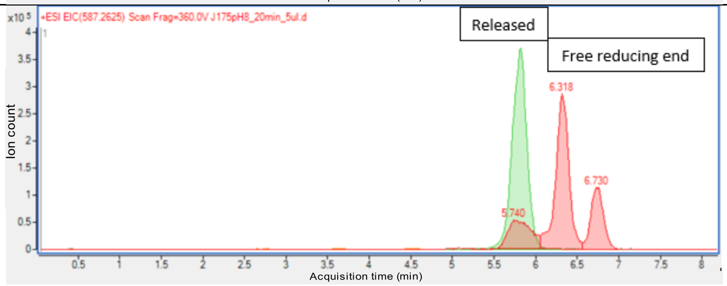
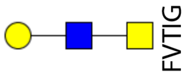	8	30min	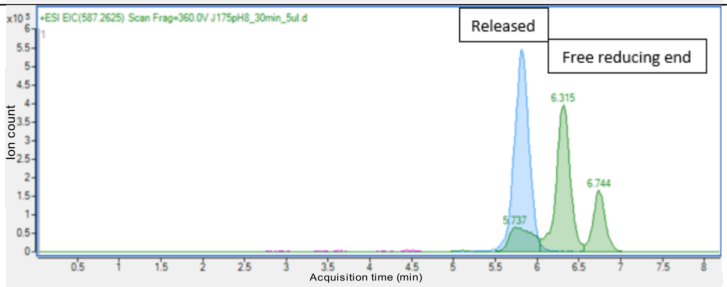
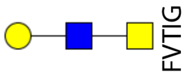	8	40 min	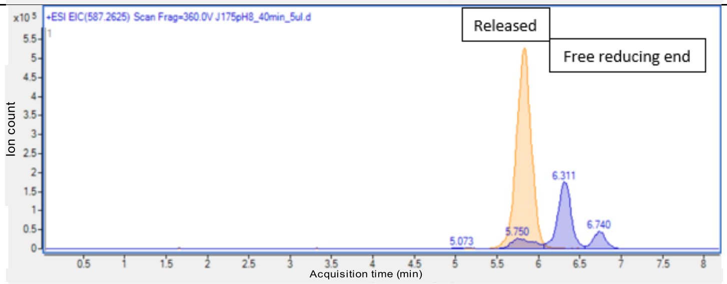

Chromatograms of peptide reaction mixtures at different time points and pH values during oxidative release.

Glycopeptide	pH of hypochlorite solution	Reaction time (min)	Extracted ion chromatogram
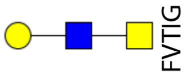	8	50 min	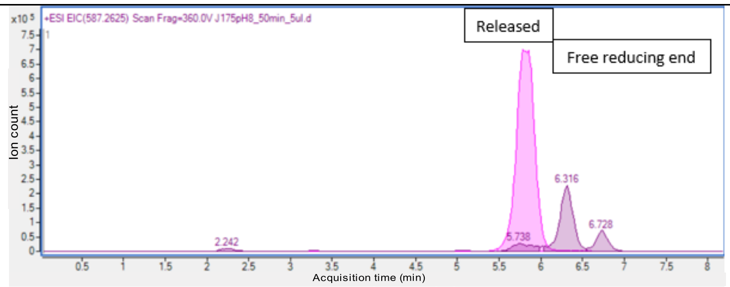
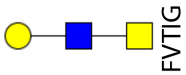	8	60 min	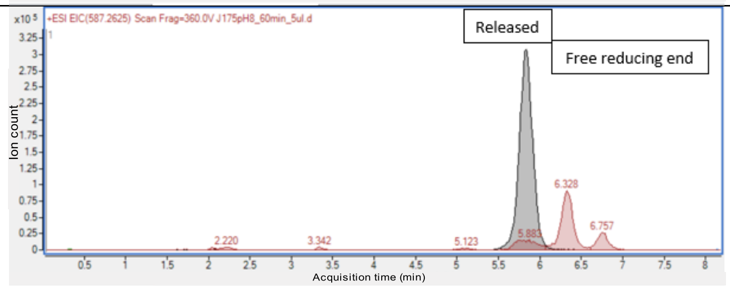
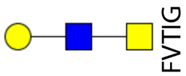	9	0 min	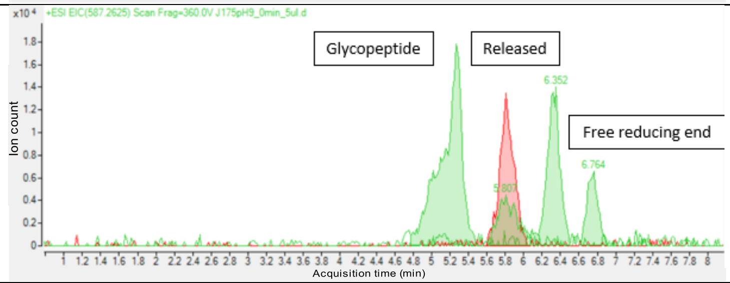
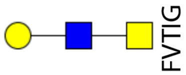	9	10 min	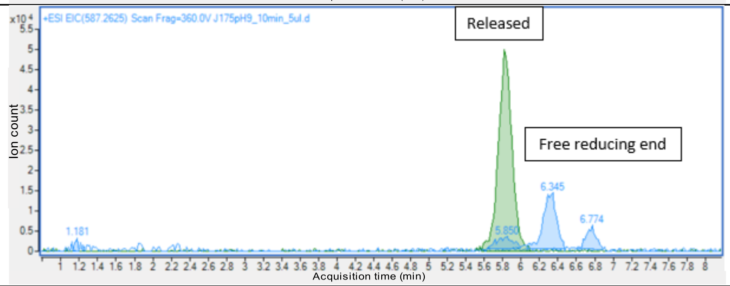
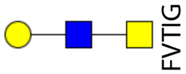	9	20 min	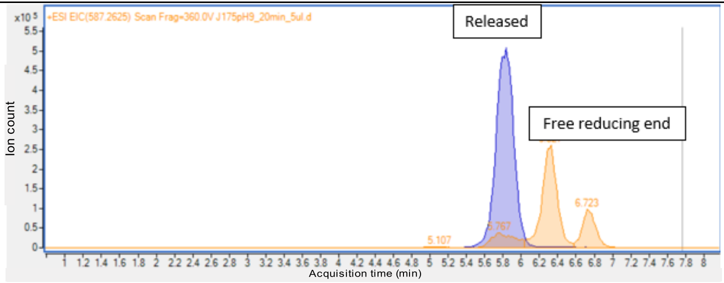

Chromatograms of peptide reaction mixtures at different time points and pH values during oxidative release.

Glycopeptide	pH of hypochlorite solution	Reaction time (min)	Extracted ion chromatogram
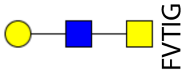	9	30min	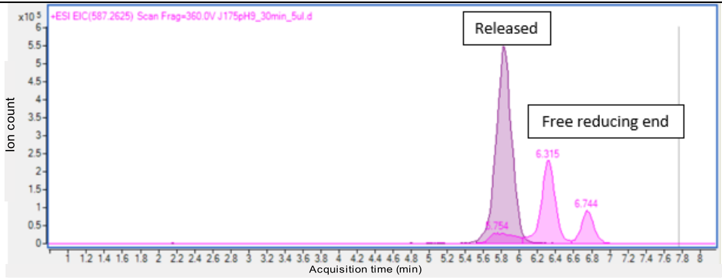
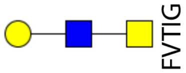	9	40 min	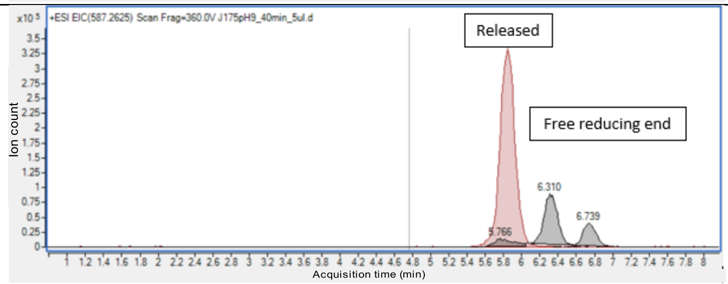
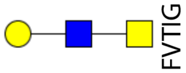	9	50 min	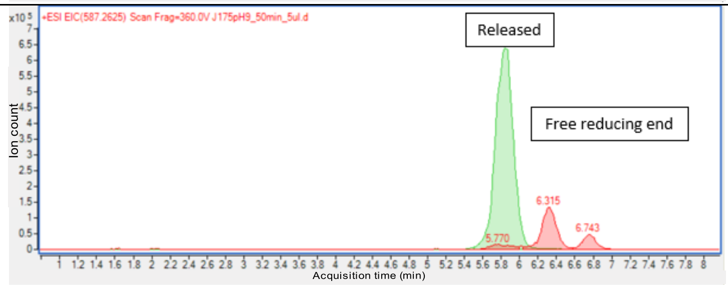
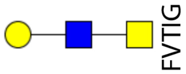	9	60 min	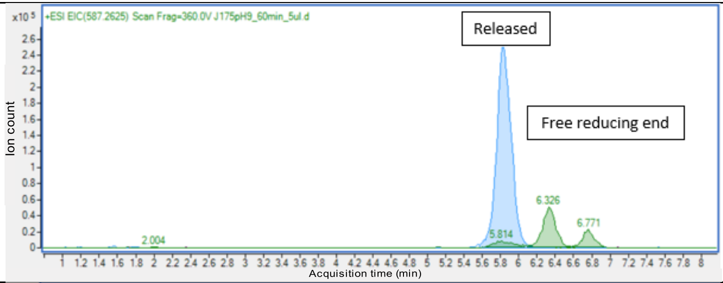
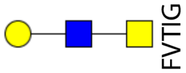	10	0 min	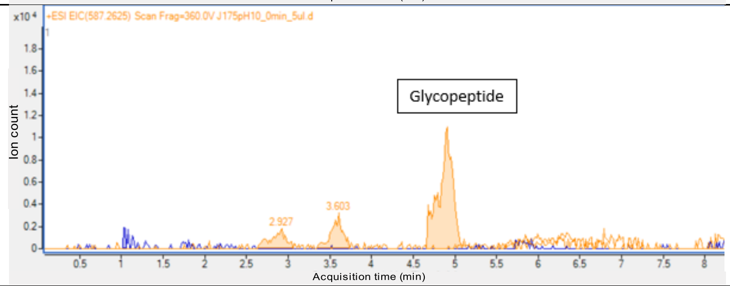

Chromatograms of peptide reaction mixtures at different time points and pH values during oxidative release.

Glycopeptide	pH of hypochlorite solution	Reaction time (min)	Extracted ion chromatogram
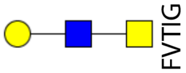	10	10 min	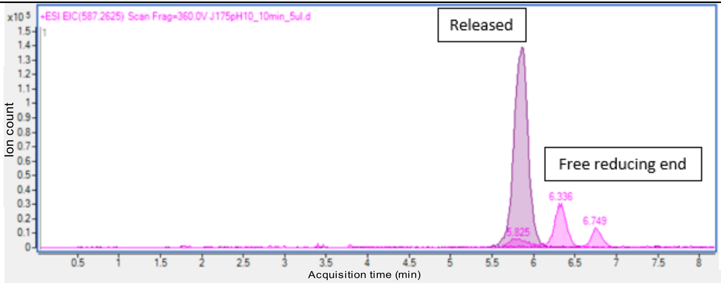
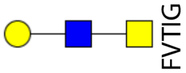	10	20 min	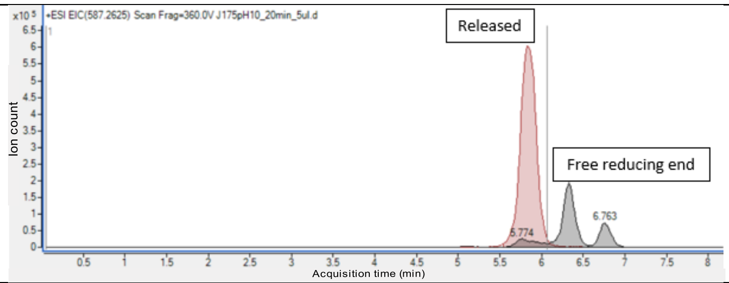
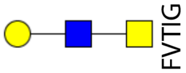	10	30min	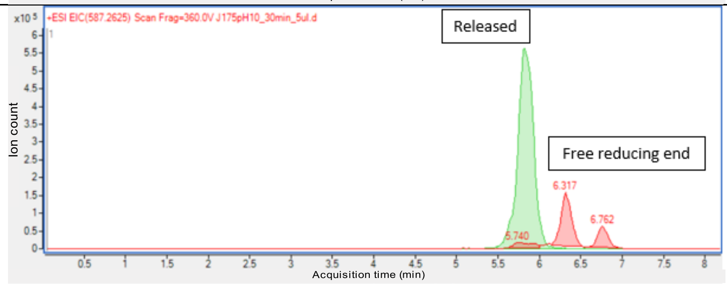
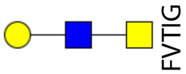	10	40 min	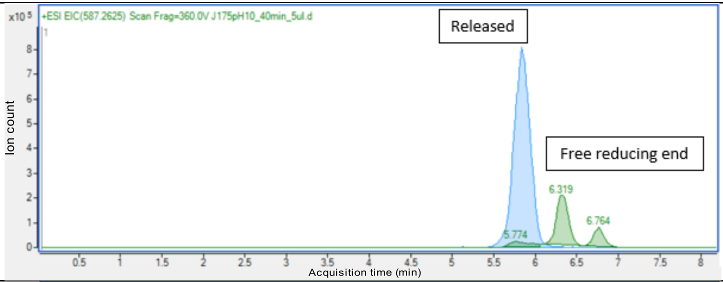
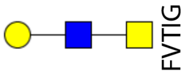	10	50 min	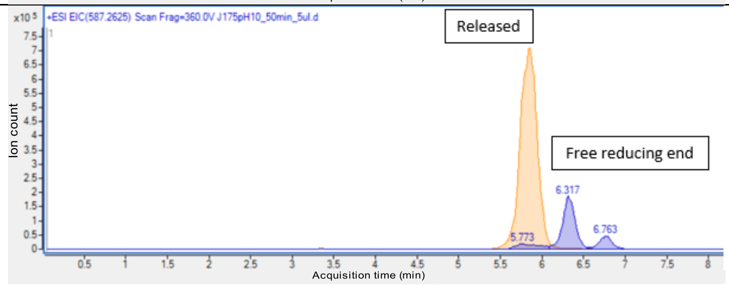

Chromatograms of peptide reaction mixtures at different time points and pH values during oxidative release.

Glycopeptide	pH of hypochlorite solution	Reaction time (min)	Extracted ion chromatogram
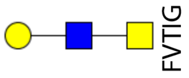	10	60 min	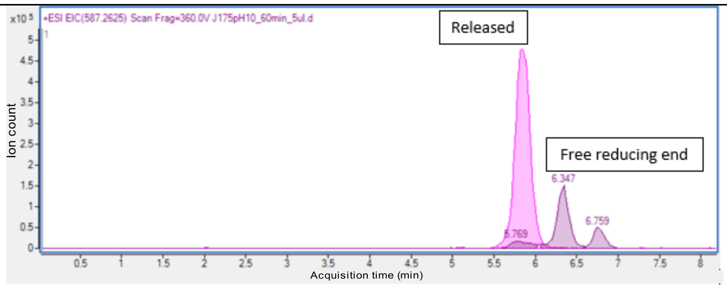
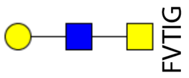	11	0 min	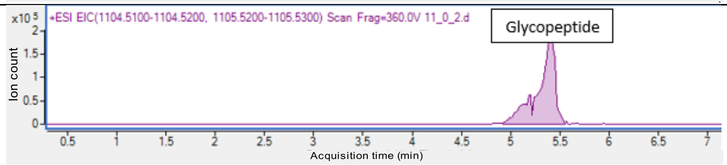
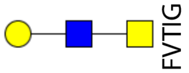	11	10 min	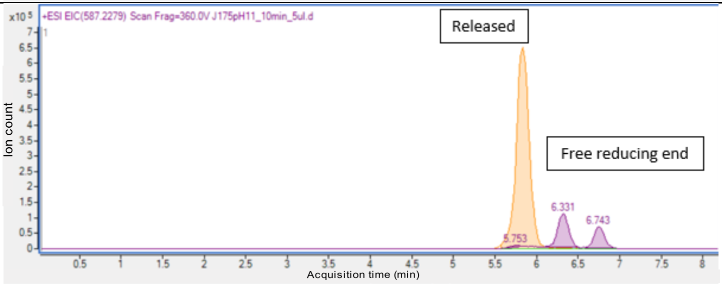
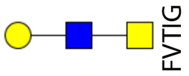	11	20 min	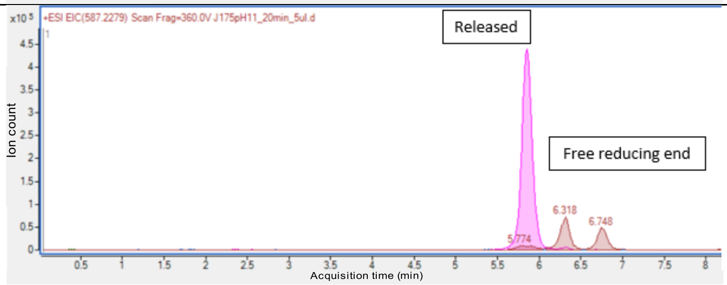
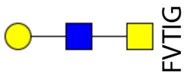	11	30min	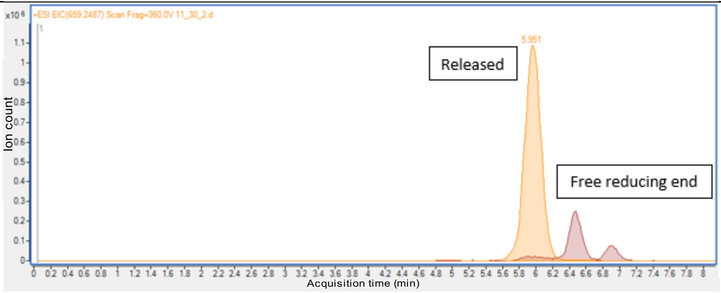
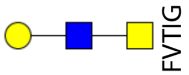	11	40 min	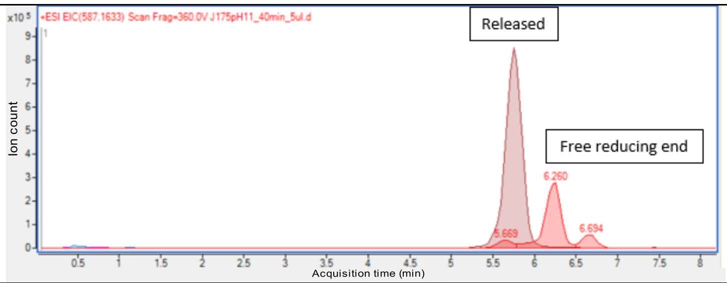

Chromatograms of peptide reaction mixtures at different time points and pH values during oxidative release.

Glycopeptide	pH of hypochlorite solution	Reaction time (min)	Extracted ion chromatogram
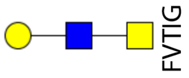	11	50 min	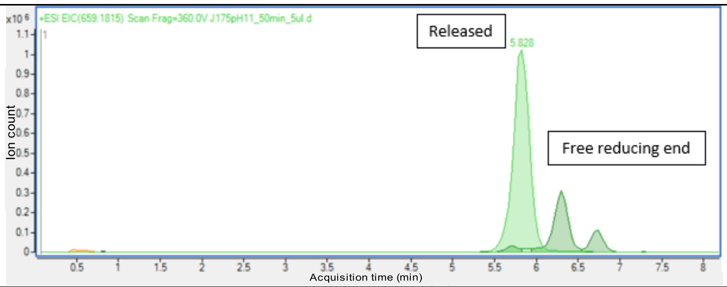
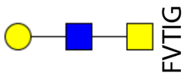	11	60 min	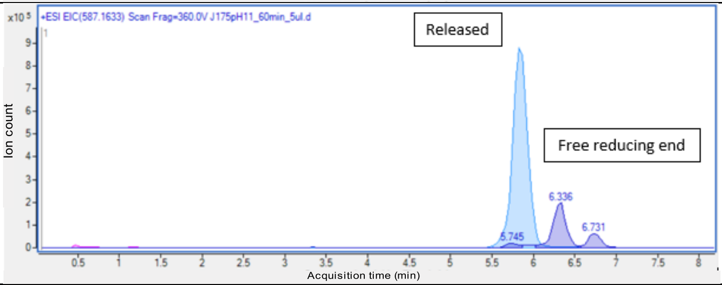
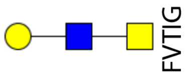	12	0 min	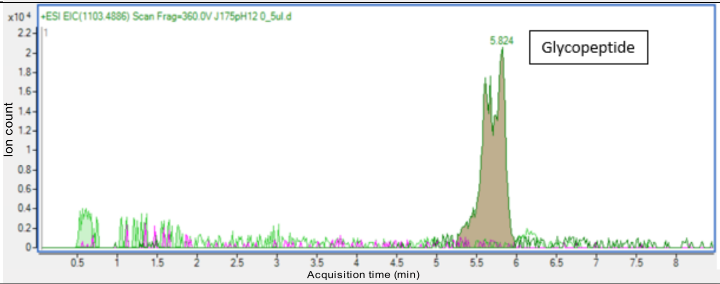
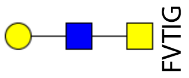	12	10 min	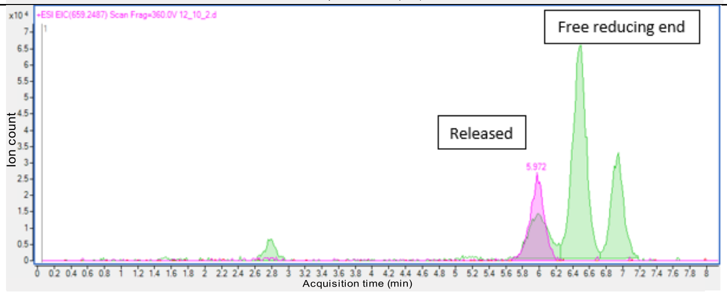
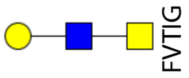	12	20 min	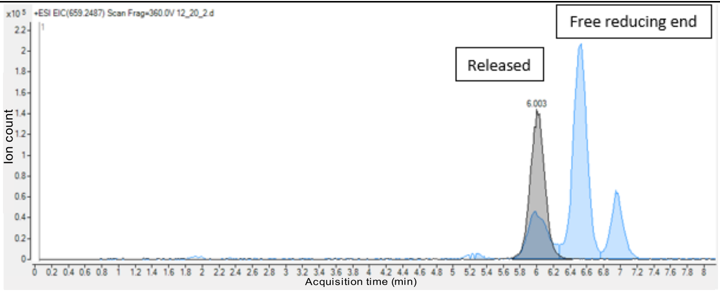

Chromatograms of peptide reaction mixtures at different time points and pH values during oxidative release.

Glycopeptide	pH of hypochlorite solution	Reaction time (min)	Extracted ion chromatogram
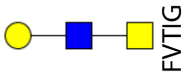	12	30min	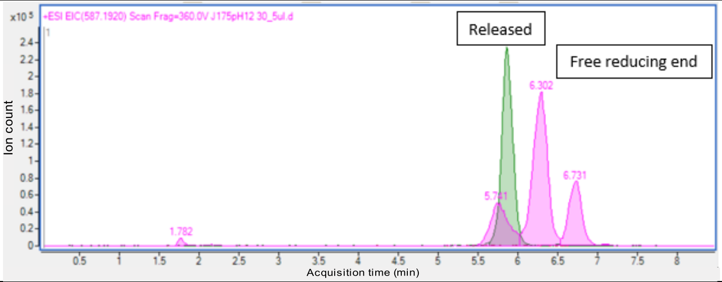
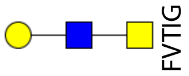	12	40 min	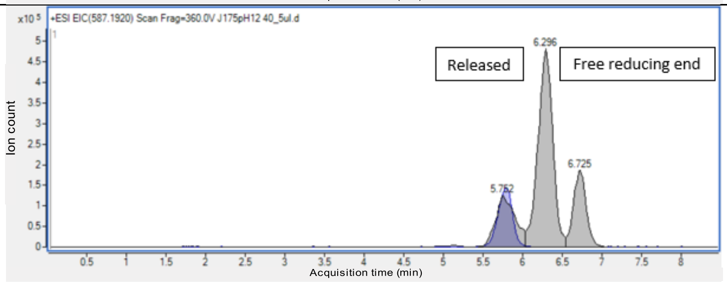
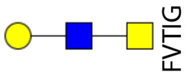	12	50 min	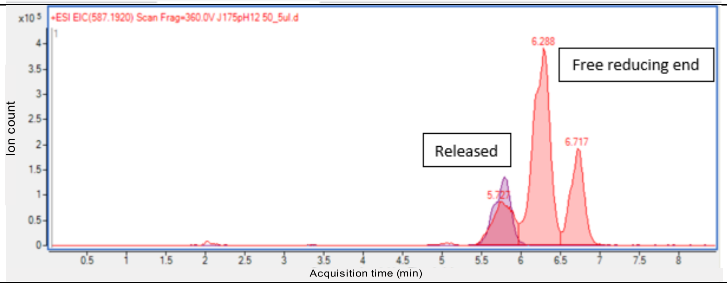
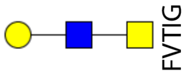	12	60 min	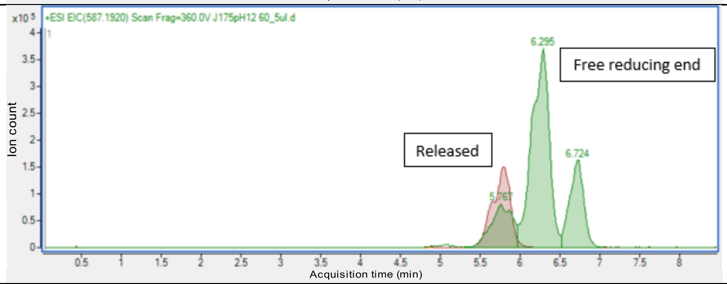
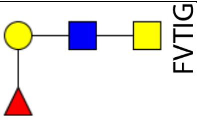	6.75	0 min	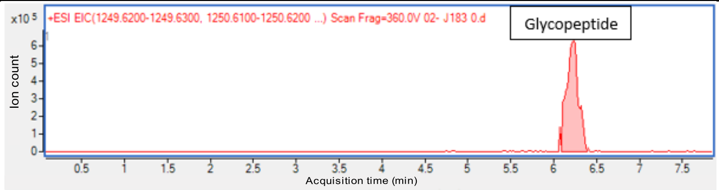
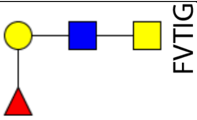	6.75	10 min	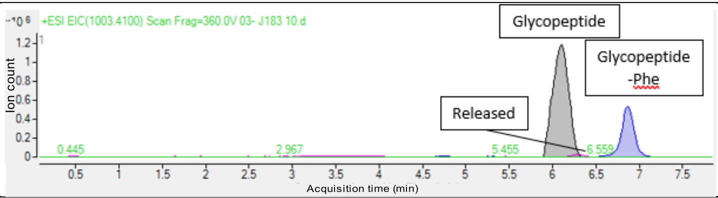

Chromatograms of peptide reaction mixtures at different time points and pH values during oxidative release.

Glycopeptide	pH of hypochlorite solution	Reaction time (min)	Extracted ion chromatogram
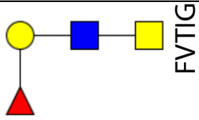	6.75	20 min	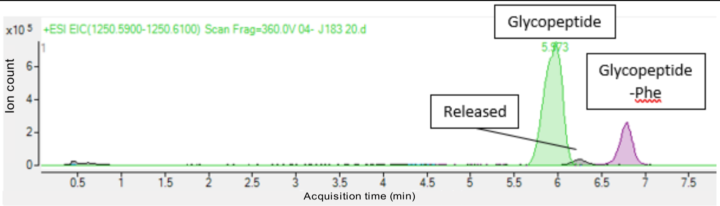
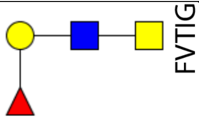	6.75	30min	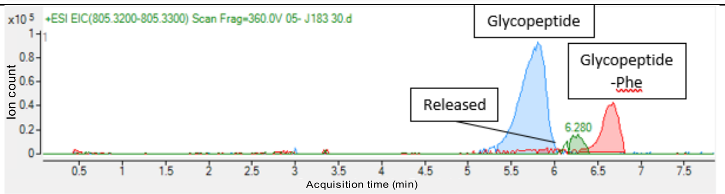
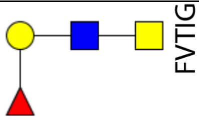	6.75	40 min	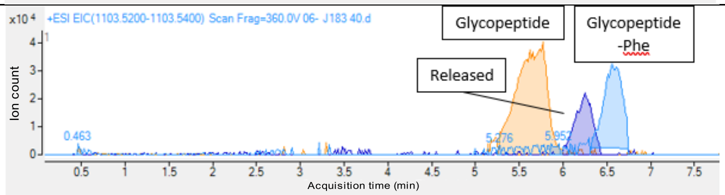
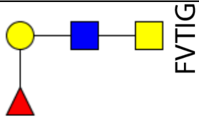	6.75	50 min	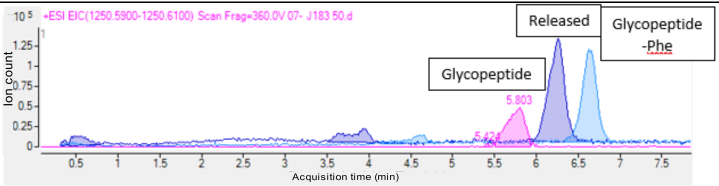
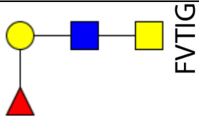	6.75	60 min	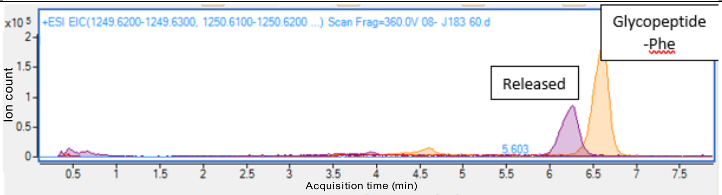
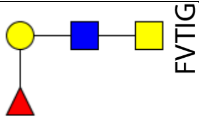	6.75	70 min	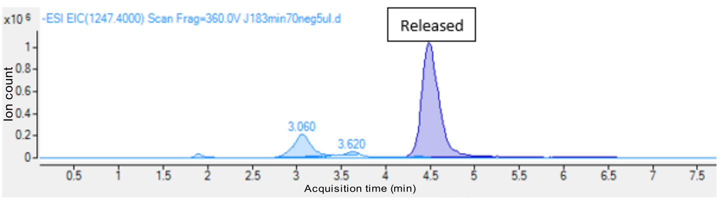
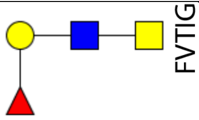	6.75	80 min	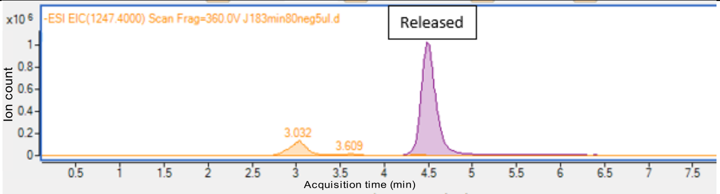
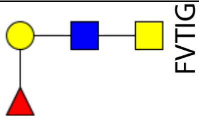	6.75	90 min	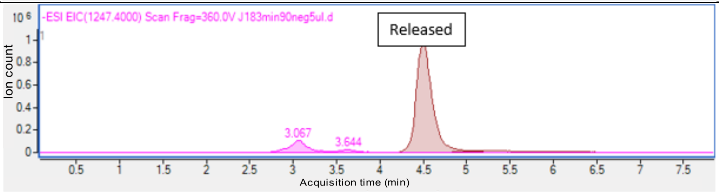

Chromatograms of peptide reaction mixtures at different time points and pH values during oxidative release.

Glycopeptide	pH of hypochlorite solution	Reaction time (min)	Extracted ion chromatogram
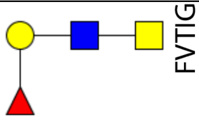	6.75	100 min	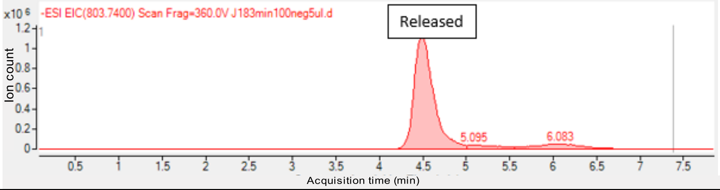
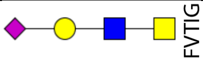	6.75	0 min	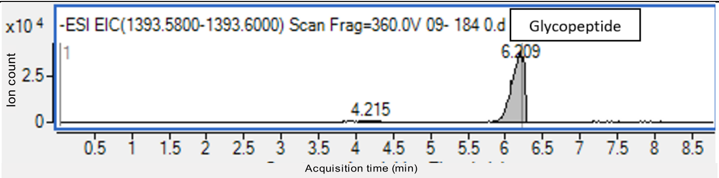
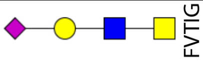	6.75	10 min	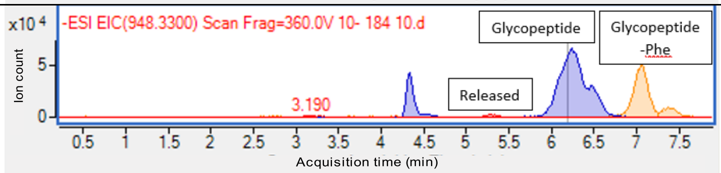
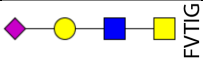	6.75	20 min	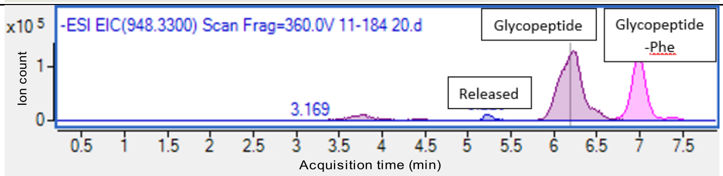
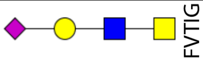	6.75	30min	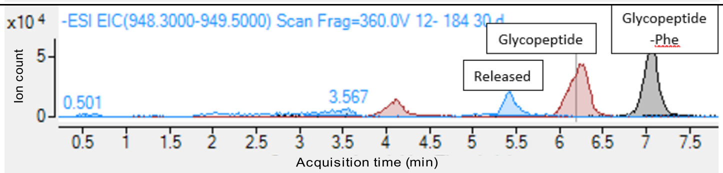
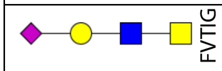	6.75	40 min	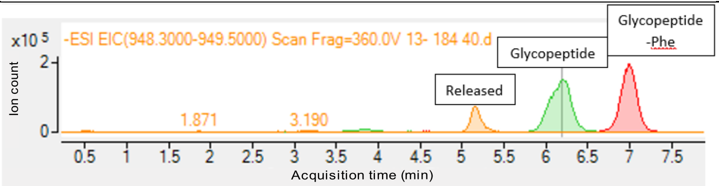
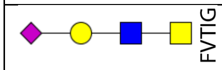	6.75	50 min	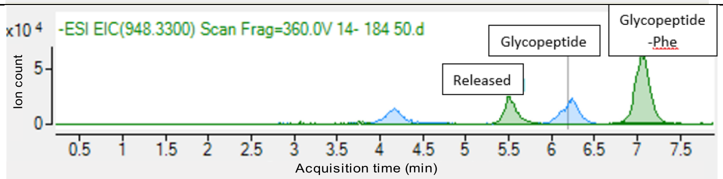
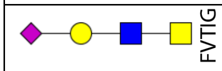	6.75	60 min	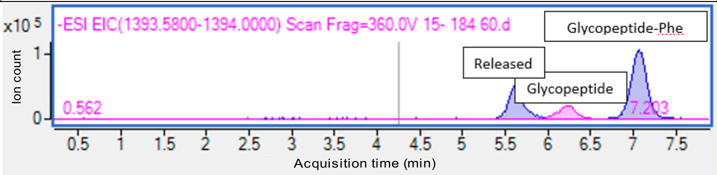

Chromatograms of peptide reaction mixtures at different time points and pH values during oxidative release.

Glycopeptide	pH of hypochlorite solution	Reaction time (min)	Extracted ion chromatogram
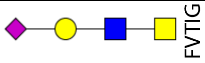	6.75	70 min	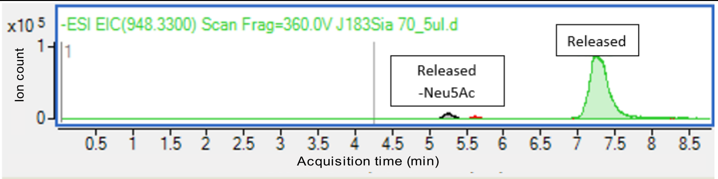
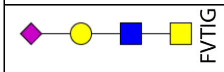	6.75	80 min	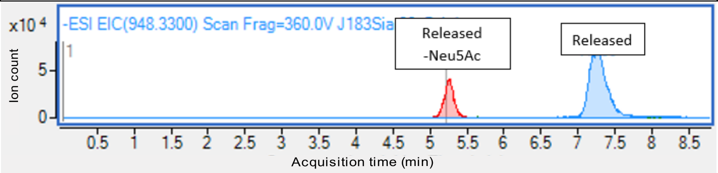
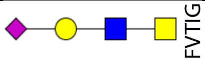	6.75	90 min	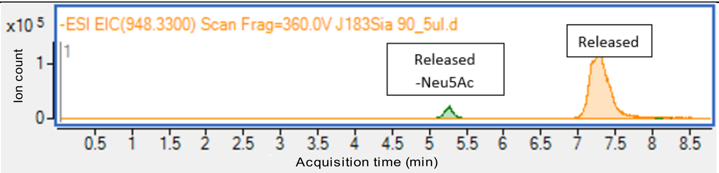
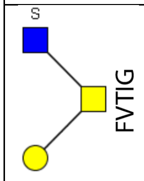	6.75	0 min	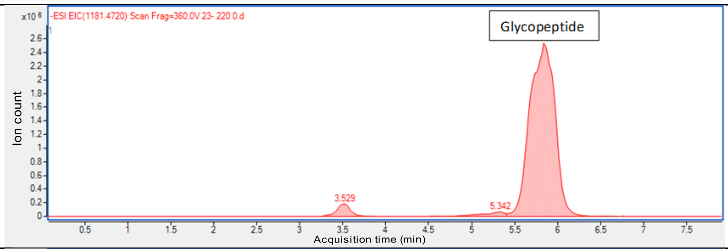
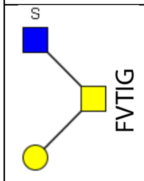	6.75	10 min	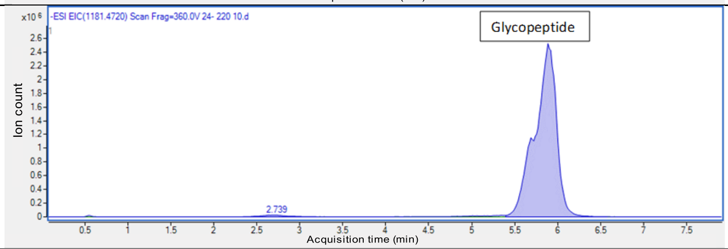
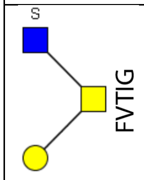	6.75	20 min	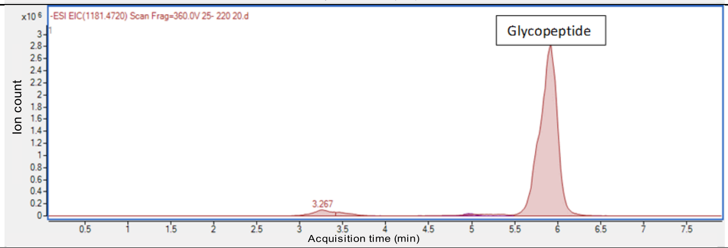
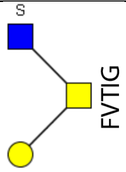	6.75	30min	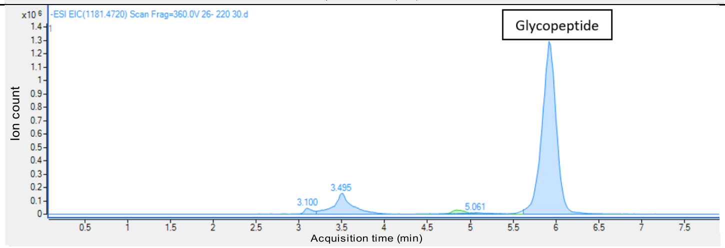

Chromatograms of peptide reaction mixtures at different time points and pH values during oxidative release.

Glycopeptide	pH of hypochlorite solution	Reaction time (min)	Extracted ion chromatogram
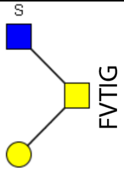	6.75	40 min	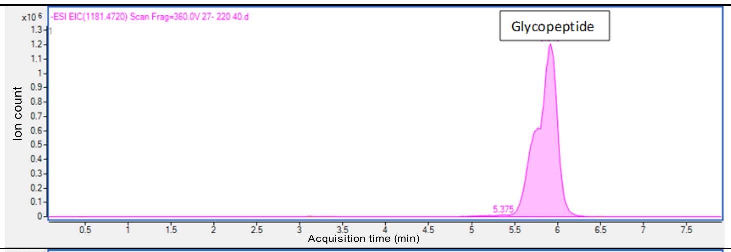
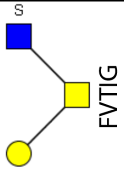	6.75	50 min	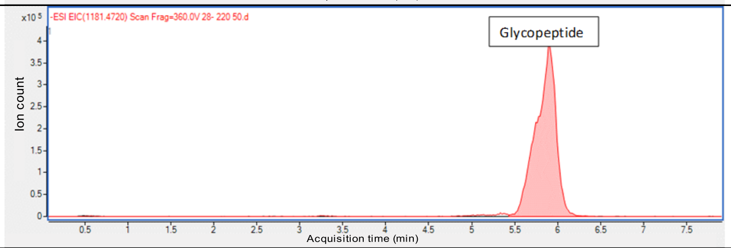
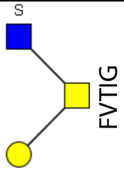	6.75	60 min	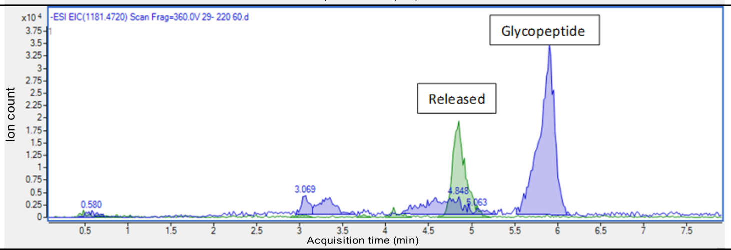
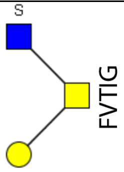	6.75	80 min	No glycan/glycopeptide observed
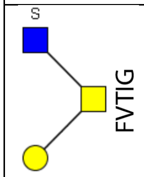	6.75	100 min	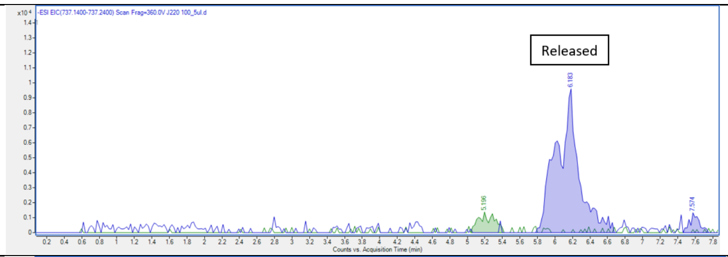
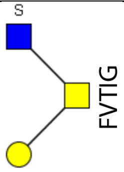	6.75	120 min	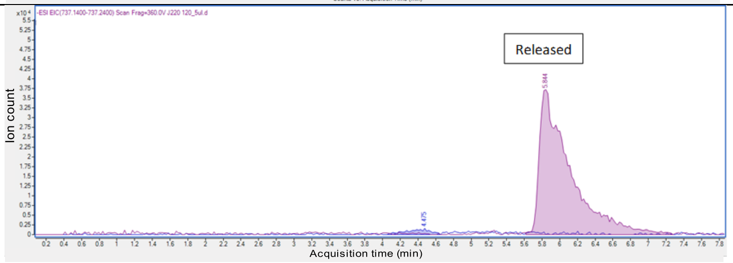

Table II: Compositions of *O*-glycans released from BSM with neutralized hypochlorite and analyzed with LC-MS with an eluent pH of 6.5. AA=amino acid (T=threonine, S=serine), Hex=hexose, Fuc=fucose, Neu5Ac= *N*-Acetylneuraminic acid, Neu5Gc= *N*-Glycolylneuraminic acid,OAc=O-acetylated, HexA=hexosamine, Sulf=sulfated.

O-glycan structures identified in BSM after oxidative release and analysis at pH 6.5.

Suggested structure	Observed *m/z* $[M-H]^-$	Calculated *m/z* $[M-H]^-$	Relative abundancy (%)	AA	Hex	HexNAc	Fuc	Neu5Ac	Neu5Gc	OAc	HexA	Sulf
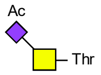	625.2105	625.2092	15.1	T	0	1	0	1	0	1	0	0
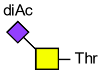	667.2211	667.2198	10.5	T	0	1	0	1	0	2	0	0
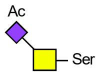	611.1948	611.1936	7.50	S	0	1	0	1	0	1	0	0
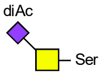	653.2053	653.2042	5.98	S	0	1	0	1	0	2	0	0
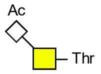	641.2055	641.2041	5.46	T	0	1	0	0	1	1	0	0
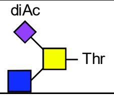	870.3014	870.2992	4.34	T	0	2	0	1	0	2	0	0
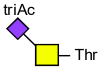	709.231	709.2304	4.09	T	0	1	0	1	0	3	0	0
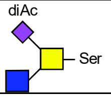	856.2857	856.2836	3.95	S	0	2	0	1	0	2	0	0
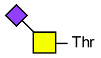	583.1997	583.1986	3.68	T	0	1	0	1	0	0	0	0
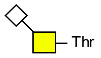	599.1947	599.1935	3.08	T	0	1	0	0	1	0	0	0
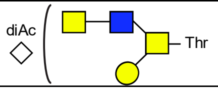	1251.4298	1251.4263	2.67	T	1	3	0	0	1	2	0	0
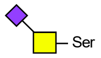	569.1838	569.1830	2.47	S	0	1	0	1	0	0	0	0
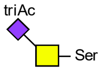	695.2161	695.2148	2.32	S	0	1	0	1	0	3	0	0
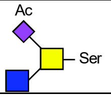	814.2747	814.2730	1.95	S	0	2	0	1	0	1	0	0
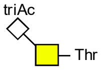	725.2264	725.2253	1.84	T	0	1	0	0	1	3	0	0
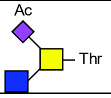	828.2906	828.2886	1.59	T	0	2	0	1	0	1	0	0
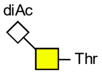	683.2161	683.2147	1.52	T	0	1	0	0	1	2	0	0

O-glycan structures identified in BSM after oxidative release and analysis at pH 6.5.

Suggested structure	Observed *m/z* [M-H]$^{-}$	Calculated *m/z* [M-H]$^{-}$	Relative abundancy (%)	AA	Hex	HexNAc	Fuc	Neu5Ac	Neu5Gc	OAc	HexA	Sulf
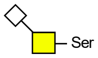	585.1788	585.1779	1.39	S	0	1	0	0	1	0	0	0
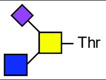	786.2796	786.2780	1.26	T	0	2	0	1	0	0	0	0
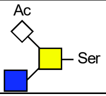	830.27	830.2679	1.19	S	0	2	0	0	1	1	0	0
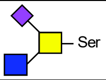	772.2643	772.2624	1.05	S	0	2	0	1	0	0	0	0
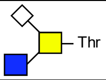	802.2748	802.2729	1.03	T	0	2	0	0	1	0	0	0
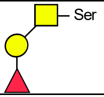	586.1991	586.1983	1.02	S	1	1	1	0	0	0	0	0
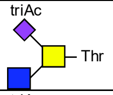	912.3121	912.3098	0.97	T	0	2	0	1	0	3	0	0
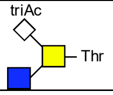	928.3072	928.3047	0.93	T	0	2	0	0	1	3	0	0
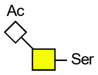	627.1902	627.1885	0.84	S	0	1	0	0	1	1	0	0
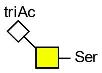	711.2111	711.2097	0.79	S	0	1	0	0	1	3	0	0
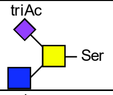	898.2968	898.2942	0.78	S	0	2	0	1	0	3	0	0
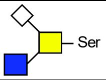	788.2593	788.2573	0.75	S	0	2	0	0	1	0	0	0
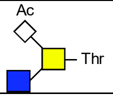	844.2858	844.2835	0.71	T	0	2	0	0	1	1	0	0
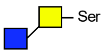	481.1677	481.1670	0.69	S	0	2	0	0	0	0	0	0
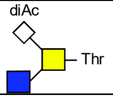	886.2968	886.2941	0.66	T	0	2	0	0	1	2	0	0
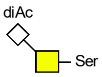	669.2008	669.1991	0.66	S	0	1	0	0	1	2	0	0
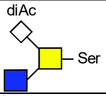	872.2809	872.2785	0.52	S	0	2	0	0	1	2	0	0
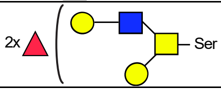	1097.3909	1097.3884	0.50	S	2	2	2	0	0	0	0	0

O-glycan structures identified in BSM after oxidative release and analysis at pH 6.5.

Suggested structure	Observed *m/z* [M-H]$^-$	Calculated *m/z* [M-H]$^-$	Relative abundancy (%)	AA	Hex	HexNAc	Fuc	Neu5Ac	Neu5Gc	OAc	HexA	Sulf
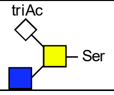	914.2915	914.2891	0.44	S	0	2	0	0	1	3	0	0
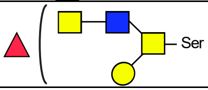	992.36	992.3571	0.37	S	1	3	1	0	0	0	0	0
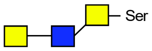	684.2477	684.2463	0.33	S	0	3	0	0	0	0	0	0
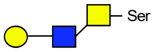	643.2211	643.2198	0.28	S	1	2	0	0	0	0	0	0
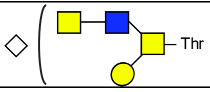	1167.4091	1167.4051	0.27	T	1	3	0	0	1	0	0	0
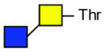	495.1836	495.1826	0.22	T	0	2	0	0	0	0	0	0
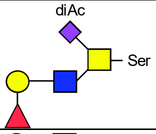	1164.3977	1164.3943	0.17	S	1	2	1	1	0	2	0	0
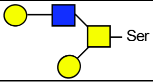	805.2743	805.2726	0.15	S	2	2	0	0	0	0	0	0
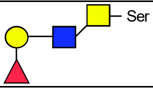	789.2789	789.2777	0.15	S	1	2	1	0	0	0	0	0
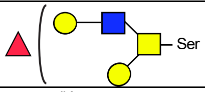	951.3324	951.3305	0.15	S	2	2	1	0	0	0	0	0
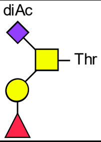	975.3333	975.3306	0.14	T	1	1	1	1	0	2	0	0
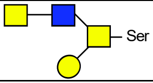	846.3009	846.2992	0.13	S	1	3	0	0	0	0	0	0
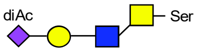	1018.3384	1018.3364	0.13	S	1	2	0	1	0	2	0	0
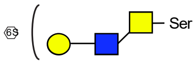	723.1776	723.1766	0.12	S	1	2	0	0	0	0	0	1
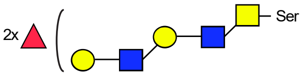	1300.471	1300.4678	0.11	S	2	3	2	0	0	0	0	0
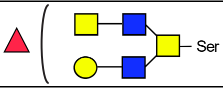	1195.4398	1195.4364	0.11	S	1	4	1	0	0	0	0	0
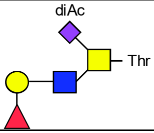	1178.4123	1178.4100	0.11	T	1	2	1	1	0	2	0	0

O-glycan structures identified in BSM after oxidative release and analysis at pH 6.5.

Suggested structure	Observed *m/z* [M-H]⁻	Calculated *m/z* [M-H]⁻	Relative abundancy (%)	AA	Hex	HexNAc	Fuc	Neu5Ac	Neu5Gc	OAc	HexA	Sulf
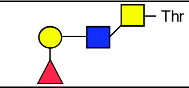	803.2954	803.2933	0.10	T	1	2	1	0	0	0	0	0
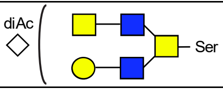	1440.4927	1440.4900	0.10	S	1	4	0	0	1	2	0	0
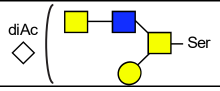	1237.4146	1237.4107	0.098	S	1	3	0	0	1	2	0	0
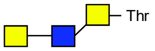	698.2635	698.2620	0.084	T	0	3	0	0	0	0	0	0
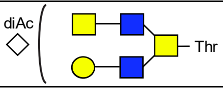	1454.5076	1454.5057	0.083	T	1	4	0	0	1	2	0	0
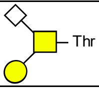	761.2478	761.2464	0.082	T	1	1	0	0	1	0	0	0
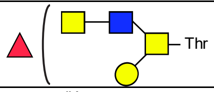	1006.3648	1006.3727	0.077	T	1	3	1	0	0	0	0	0
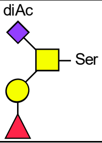	961.3174	961.3149	0.077	S	1	1	1	1	0	2	0	0
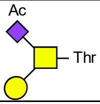	787.2627	787.2621	0.076	T	1	1	0	1	0	1	0	0
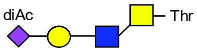	1032.353	1032.3520	0.073	T	1	2	0	1	0	2	0	0
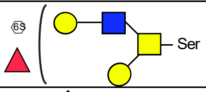	1031.2891	1031.2873	0.069	S	2	2	1	0	0	0	0	1
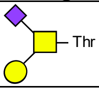	745.2523	745.2515	0.066	T	1	1	0	1	0	0	0	0
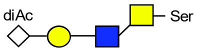	1034.3325	1034.3313	0.064	S	1	2	0	0	1	2	0	0
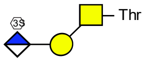	710.1433	710.1450	0.064	T	1	1	0	0	0	0	1	1
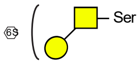	520.0981	520.0972	0.060	S	1	1	0	0	0	0	0	1
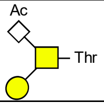	803.2581	803.2570	0.057	T	1	1	0	0	1	1	0	0
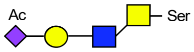	976.3271	976.3258	0.055	S	1	2	0	1	0	1	0	0

O-glycan structures identified in BSM after oxidative release and analysis at pH 6.5.

Suggested structure	Observed *m/z* [M-H]⁻	Calculated *m/z* [M-H]⁻	Relative abundancy (%)	AA	Hex	HexNAc	Fuc	Neu5Ac	Neu5Gc	OAc	HexA	Sulf
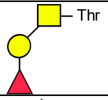	600.2144	600.2140	0.054	T	1	1	1	0	0	0	0	0
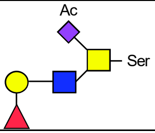	1122.3852	1122.3837	0.053	S	1	2	1	1	0	1	0	0
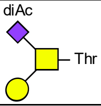	829.2739	829.2727	0.051	T	1	1	0	1	0	2	0	0
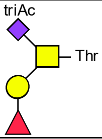	1017.3435	1017.3412	0.046	T	1	1	1	1	0	3	0	0
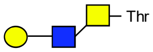	657.2359	657.2354	0.044	T	1	2	0	0	0	0	0	0
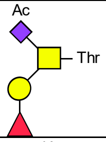	933.3217	933.3200	0.041	T	1	1	1	1	0	1	0	0
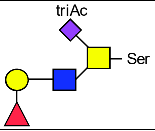	1206.4056	1206.4049	0.039	S	1	2	1	1	0	3	0	0
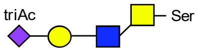	1060.3482	1060.3470	0.038	S	1	2	0	1	0	3	0	0
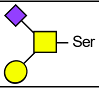	731.2363	731.2358	0.037	S	1	1	0	1	0	0	0	0
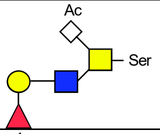	1138.3802	1138.3786	0.036	S	1	2	1	0	1	1	0	0
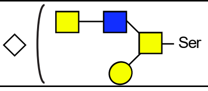	1153.393	1153.3895	0.036	S	1	3	0	0	1	0	0	0
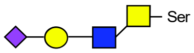	934.316	934.3152	0.034	S	1	2	0	1	0	0	0	0
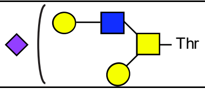	1110.3849	1110.3837	0.032	T	2	2	0	1	0	0	0	0
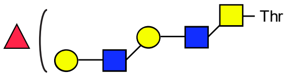	1168.4214	1168.4255	0.031	T	2	3	1	0	0	0	0	0
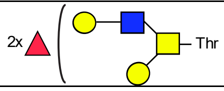	1111.4054	1111.4041	0.029	T	2	2	2	0	0	0	0	0

O-glycan structures identified in BSM after oxidative release and analysis at pH 6.5.

Suggested structure	Observed *m/z* $[M-H]^-$	Calculated *m/z* $[M-H]^-$	Relative abundancy (%)	AA	Hex	HexNAc	Fuc	Neu5Ac	Neu5Gc	OAc	HexA	Sulf
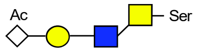	992.3218	992.3207	0.029	S	1	2	0	0	1	1	0	0
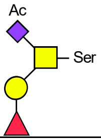	919.3063	919.3043	0.027	S	1	1	1	1	0	1	0	0
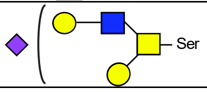	1096.3694	1096.3680	0.027	S	2	2	0	1	0	0	0	0
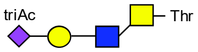	1074.3639	1074.3626	0.027	T	1	2	0	1	0	3	0	0
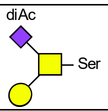	815.2582	815.2570	0.027	S	1	1	0	1	0	2	0	0
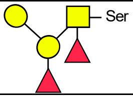	894.312	894.3090	0.025	S	2	1	2	0	0	0	0	0
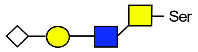	950.3121	950.3101	0.025	S	1	2	0	0	1	0	0	0
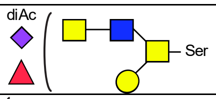	1367.475	1367.4737	0.024	S	1	3	1	1	0	2	0	0
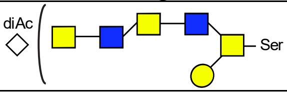	1643.5718	1643.5694	0.024	S	1	5	0	0	1	2	0	0
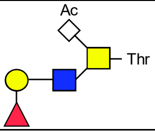	1152.3955	1152.3942	0.023	T	1	2	1	0	1	1	0	0
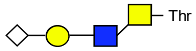	964.3268	964.3257	0.023	T	1	2	0	0	1	0	0	0
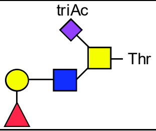	1220.4209	1220.4206	0.024	T	1	2	1	1	0	3	0	0
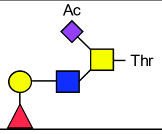	1136.4007	1136.3994	0.023	T	1	2	1	1	0	1	0	0
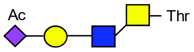	990.3423	990.3414	0.022	T	1	2	0	1	0	1	0	0
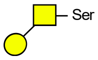	440.1406	440.1404	0.020	S	1	1	0	0	0	0	0	0

O-glycan structures identified in BSM after oxidative release and analysis at pH 6.5.

Suggested structure	Observed *m/z* [M-H]⁻	Calculated *m/z* [M-H]⁻	Relative abundancy (%)	AA	Hex	HexNAc	Fuc	Neu5Ac	Neu5Gc	OAc	HexA	Sulf
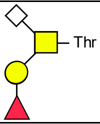	907.3058	907.3043	0.020	T	1	1	1	0	1	0	0	0
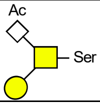	789.2433	789.2413	0.019	S	1	1	0	0	1	1	0	0
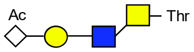	1006.3378	1006.3363	0.019	T	1	2	0	0	1	1	0	0
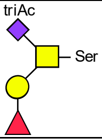	1003.3272	1003.3255	0.019	S	1	1	1	1	0	3	0	0
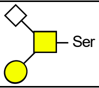	747.2309	747.2307	0.019	S	1	1	0	0	1	0	0	0
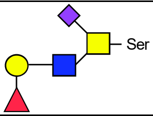	1080.3735	1080.3731	0.019	S	1	2	1	1	0	0	0	0
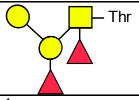	908.3265	908.3247	0.018	T	2	1	2	0	0	0	0	0
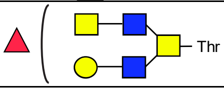	1209.4533	1209.4521	0.018	T	1	4	1	0	0	0	0	0
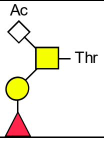	949.3164	949.3149	0.017	T	1	1	1	0	1	1	0	0
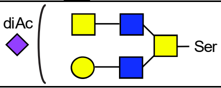	1424.4936	1424.4951	0.016	S	1	4	0	1	0	2	0	0
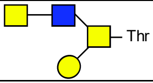	860.3152	860.3148	0.016	T	1	3	0	0	0	0	0	0
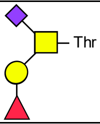	891.3102	891.3094	0.016	T	1	1	1	1	0	0	0	0
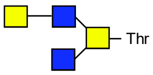	901.3424	901.3413	0.016	T	0	4	0	0	0	0	0	0
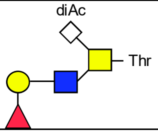	1194.4035	1194.4048	0.015	T	1	2	1	0	1	2	0	0

O-glycan structures identified in BSM after oxidative release and analysis at pH 6.5.

Suggested structure	Observed m/z $[M-H]^-$	Calculated m/z $[M-H]^-$	Relative abundancy (%)	AA	Hex	HexNAc	Fuc	Neu5Ac	Neu5Gc	OAc	HexA	Sulf
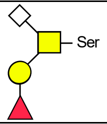	893.29	893.2886	0.015	S	1	1	1	0	1	0	0	0
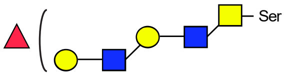	1154.411	1154.4099	0.015	S	2	3	1	0	0	0	0	0
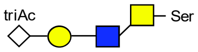	1076.3427	1076.3419	0.015	S	1	2	0	0	1	3	0	0
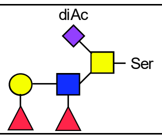	1310.4546	1310.4522	0.014	S	1	2	2	1	0	2	0	0
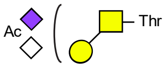	1094.3536	1094.3524	0.014	T	1	1	0	1	1	1	0	0
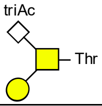	887.2808	887.2782	0.012	T	1	1	0	0	1	3	0	0
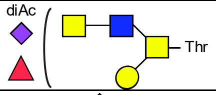	1381.4892	1381.4893	0.012	T	1	3	1	1	0	2	0	0
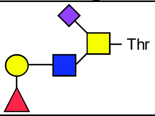	1094.3883	1094.3888	0.012	T	1	2	1	1	0	0	0	0
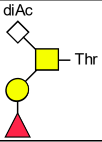	991.3266	991.3255	0.011	T	1	1	1	0	1	2	0	0
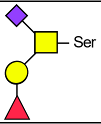	877.2949	877.2937	0.011	S	1	1	1	1	0	0	0	0
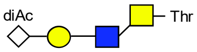	1048.3489	1048.3469	0.011	T	1	2	0	0	1	2	0	0
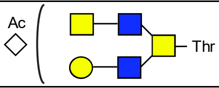	1412.4957	1412.4951	0.011	T	1	4	0	0	1	1	0	0
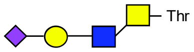	948.332	948.3308	0.011	T	1	2	0	1	0	0	0	0
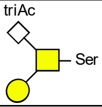	873.2608	873.2625	0.010	S	1	1	0	0	1	3	0	0
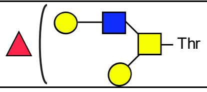	965.3472	965.3462	0.010	T	2	2	1	0	0	0	0	0

O-glycan structures identified in BSM after oxidative release and analysis at pH 6.5.

Suggested structure	Observed *m/z* [M-H]⁻	Calculated *m/z* [M-H]⁻	Relative abundancy (%)	AA	Hex	HexNAc	Fuc	Neu5Ac	Neu5Gc	OAc	HexA	Sulf
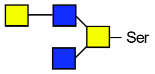	887.3264	887.3257	0.0099	S	0	4	0	0	0	0	0	0
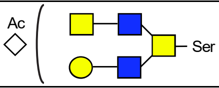	1398.4803	1398.4794	0.0097	S	1	4	0	0	1	1	0	0
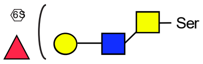	869.2373	869.2345	0.0095	S	1	2	1	0	0	0	0	1
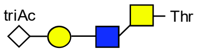	1090.3589	1090.3575	0.0095	T	1	2	0	0	1	3	0	0
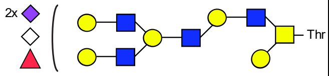	1291.9372 [M-2H]2-	2584.8917	0.0092	T	4	4	1	2	1	0	0	0
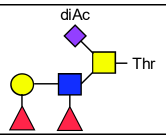	1324.4697	1324.4679	0.0088	T	1	2	2	1	0	2	0	0
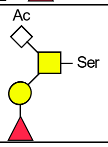	935.3001	935.2992	0.0086	S	1	1	1	0	1	1	0	0
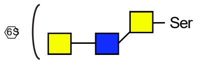	764.2051	764.2031	0.0086	S	0	3	0	0	0	0	0	1
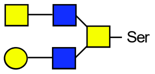	1049.3795	1049.3785	0.0084	S	1	4	0	0	0	0	0	0
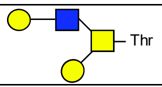	819.2885	819.2883	0.0083	T	2	2	0	0	0	0	0	0
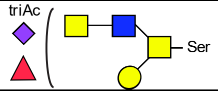	704.2403 [M-2H]2-	704.2385 [M-2H]2-	0.0082	S	1	3	1	1	0	3	0	0
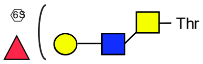	883.251	883.2501	0.0079	T	1	2	1	0	0	0	0	1
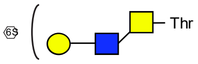	737.1926	737.1922	0.0076	T	1	2	0	0	0	0	0	1
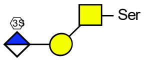	696.1263	696.1293	0.0066	S	1	1	0	0	0	0	1	1
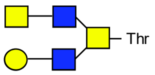	1063.3954	1063.3942	0.0063	T	1	4	0	0	0	0	0	0
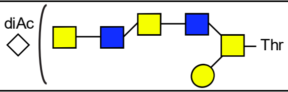	1657.5857	1657.5850	0.0058	T	1	5	0	0	1	2	0	0
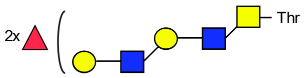	1314.4854	1314.4834	0.0057	T	2	3	2	0	0	0	0	0

O-glycan structures identified in BSM after oxidative release and analysis at pH 6.5.

Suggested structure	Observed *m/z* [M-H]⁻	Calculated *m/z* [M-H]⁻	Relative abundancy (%)	AA	Hex	HexNAc	Fuc	Neu5Ac	Neu5Gc	OAc	HexA	Sulf
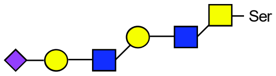	649.22 [M-2H]2-	649.2201 [M-2H]2-	0.0056	S	2	3	0	1	0	0	0	0
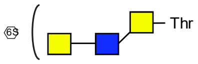	778.2194	778.2188	0.0052	T	0	3	0	0	0	0	0	1
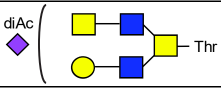	718.7516 [M-2H]2-	718.7518 [M-2H]2-	0.0052	T	1	4	0	1	0	2	0	0
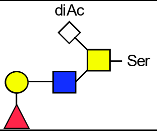	1180.391	1180.3892	0.0050	S	1	2	1	0	1	2	0	0
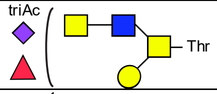	711.2465 [M-2H]2-	711.2463 [M-2H]2-	0.0043	T	1	3	1	1	0	3	0	0
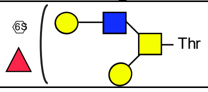	1045.3048	1045.3030	0.0042	T	2	2	1	0	0	0	0	1
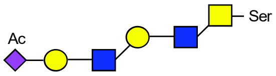	670.2241 [M-2H]2-	670.2254 [M-2H]2-	0.0039	S	2	3	0	1	0	1	0	0
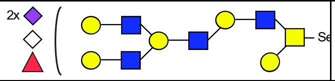	1284.9305 [M-2H]2-	1284.934 [M-2H]2-	0.0039	S	4	4	1	2	1	0	0	0
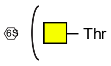	372.0602	372.0600	0.0034	T	0	1	0	0	0	0	0	1
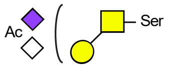	1080.3393	1080.3367	0.0034	S	1	1	0	1	1	1	0	0
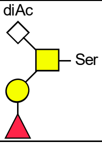	977.3106	977.3098	0.0031	S	1	1	1	0	1	2	0	0
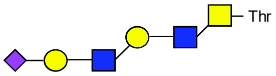	1313.4643	1313.4630	0.0029	T	2	3	0	1	0	0	0	0
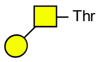	454.1563	454.1561	0.0028	T	1	1	0	0	0	0	0	0
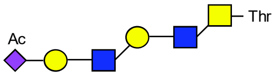	677.2337 [M-2H]2-	677.2332 [M-2H]2-	0.0021	T	2	3	0	1	0	1	0	0
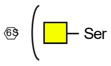	358.0443	358.0444	0.0019	S	0	1	0	0	0	0	0	1
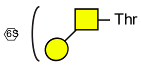	534.1141	534.1129	0.0013	T	1	1	0	0	0	0	0	1
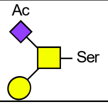	773.2485	773.2464	0.00093	S	1	1	0	1	0	1	0	0

Table III: Compositions of *O*-glycans released from BSM with reductive beta elimination and analyzed with LC-MS with an eluent pH of 6.5. Hex=hexose, Fuc=fucose, Neu5Ac= *N*-Acetylneuraminic acid, Neu5Gc= *N*-Glycolylneuraminic acid, HexA=hexosamine, Sulf=sulfate.

O-glycan structures identified in BSM after beta elimination and analysis at pH 6.5.

Suggested structure	Observed *m/z* [M-H]$^-$	Calculated *m/z* [M-H]$^-$	Retention time (min)	Relative abundancy (%)	Hex	HexNAc	Fuc	Neu5Ac	Neu5Gc	Sulf
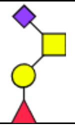	821.3057	821.3045	14.1	26.2	1	1	1	1	0	0
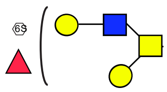	975.2992	975.2981	14.5	18.8	2	2	1	0	0	1
	975.2980		15.4	0.22						
	975.2975		15.7	0.12						
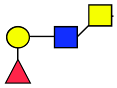	733.2895	733.2884	19.0	9.95	1	2	1	0	0	0
	733.2880		17.3	0.14						
	733.2884		20.2	0.06						
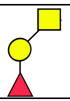	530.2093	530.2090	15.9	7.39	1	1	1	0	0	0
	530.2084		17.6	0.07						
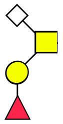	837.3007	837.2994	15.3	6.98	1	1	1	0	1	0
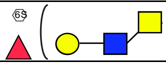	813.2461	813.2452	13.6	6.61	1	2	1	0	0	1
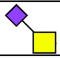	513.1938	513.1937	10.2	5.02	0	1	0	1	0	0
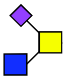	716.2741	716.2731	13.0	2.30	0	2	0	1	0	0
	716.2740		13.6	1.50						
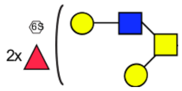	1121.3572	1121.3560	16.0	2.47	2	2	2	0	0	1
	1121.3550		16.5	0.18						
	1121.3553		16.9	0.07						
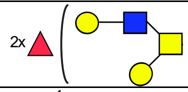	1041.4005	1041.3992	21.2	1.94	2	2	2	0	0	0
	1041.3990		21.7	0.23						
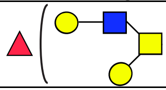	895.3428	895.3412	20.6	1.80	2	2	1	0	0	0
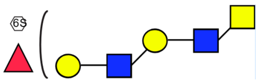	1178.3777	1178.3774	16.7	1.11	2	3	1	0	0	1
	1178.3767		16.4	0.46						
	1178.3759		17.4	0.06						
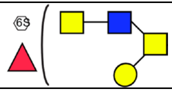	1016.3256	1016.3246	14.3	1.13	1	3	1	0	0	1
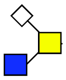	732.2686	732.2680	14.3	0.35	0	2	0	0	1	0
	732.2679		14.8	0.27						

O-glycan structures identified in BSM after beta elimination and analysis at pH 6.5.

Suggested structure	Observed *m/z* [M-H]⁻	Calculated *m/z* [M-H]⁻	Retention time (min)	Relative abundancy (%)	Hex	HexNAc	Fuc	Neu5Ac	Neu5Gc	Sulf
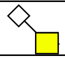	529.1889	529.1886	12.2	0.53	0	1	0	0	1	0
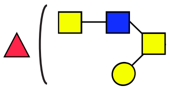	936.3683	936.3678	21.1	0.38	1	3	1	0	0	0
	936.3676		20.7	0.07						
	936.3676		20.3	0.03						
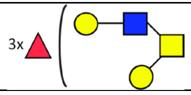	1187.4573	1187.4571	22.3	0.47	2	2	3	0	0	0
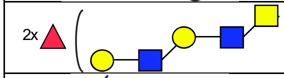	1244.4780	1244.4785	21.9	0.42	2	3	2	0	0	0
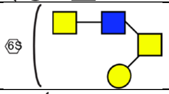	870.2669	870.2667	14.2	0.37	1	3	0	0	0	1
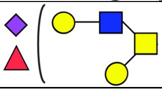	1186.4358	1186.4367	16.7	0.21	2	2	1	1	0	0
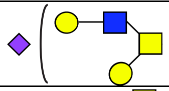	1040.3782	1040.3788	17.1	0.16	2	2	0	1	0	0
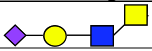	878.3257	878.3259	15.7	0.12	1	2	0	1	0	0
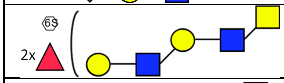	1324.4335	1324.4353	17.5	0.08	2	3	2	0	0	1
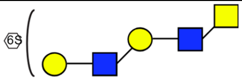	1032.3193	1032.3195	16.2	0.06	2	3	0	0	0	1
	1032.3186		15.5	0.02						
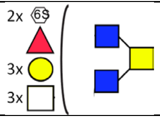	1014.3072 (as [M-2H]$^{2-}$)	1014.3090 (as [M-2H]$^{2-}$)	17.4	0.07	3	6	1	0	0	2
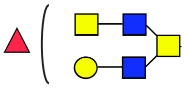	1139.4461	1139.4472	22.7	0.07	1	4	1	0	0	0
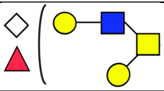	1202.4309	1202.4316	17.5	0.06	2	2	1	0	1	0
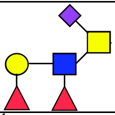	1170.4412	1170.4417	17.5	0.05	1	2	2	1	0	0
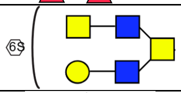	1073.3453	1073.3461	16.6	0.05	1	4	0	0	0	1
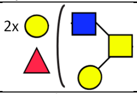	1057.3933	1057.3941	22.1	0.05	3	2	1	0	0	0
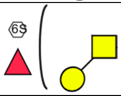	610.1654	610.1659	10.5	0.05	1	1	1	0	0	1
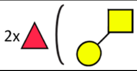	676.2665	676.2670	19.6	0.04	1	1	2	0	0	0

O-glycan structures identified in BSM after beta elimination and analysis at pH 6.5.

Suggested structure	Observed *m/z* [M-H]$^-$	Calculated *m/z* [M-H]$^-$	Retention time (min)	Relative abundancy (%)	Hex	HexNAc	Fuc	Neu5Ac	Neu5Gc	Sulf
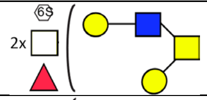	690.2249 (as [M-2H]$^{2-}$)	690.2248 (as [M-2H]$^{2-}$)	18.2	0.04	2	4	1	0	0	1
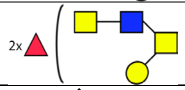	1082.4248	1082.4257	22.3	0.04	1	3	2	0	0	0
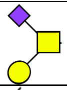	675.2458	675.2466	14.0	0.03	1	1	0	1	0	0
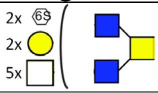	1063.3324 [M-2H]$^{2-}$	1063.3330 (as [M-2H]$^{2-}$)	20.1	0.03	2	8	0	0	0	2
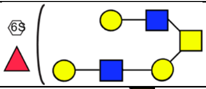	669.7114 (as [M-2H]$^{2-}$)	669.7115 (as [M-2H]$^{2-}$)	17.6	0.03	3	3	1	0	0	1
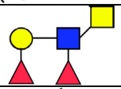	879.3459	879.3463	19.3	0.03	1	2	2	0	0	0
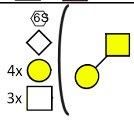	1013.8167 (as [M-2H]$^{2-}$)	1013.8202 (as [M-2H]$^{2-}$)	20.9	0.02	5	4	0	0	1	1
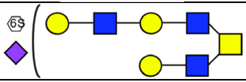	843.7713 (as [M-2H]$^{2-}$)	843.7699 (as [M-2H]$^{2-}$)	16.6	0.02	3	4	0	1	0	1

Table IV: Compositions of *O*-glycans released from BSM with reductive beta elimination and analyzed with LC-MS with an eluent pH of 7.8. Hex=hexose, Fuc=fucose, Neu5Ac= *N*-Acetylneuraminic acid, Neu5Gc= *N*-Glycolylneuraminic acid, HexA=hexosamine, Sulf=sulfate.

O-glycan structures identified in BSM after beta elimination and analysis at pH 7.8.

Composition	Observed m/z $[M-H]^-$	Calculated m/z $[M-H]^-$	Retention time (min)	Relative abundancy (%)	Hex	HexNAc	Fuc	Neu5Ac	Neu5Gc	Sulf
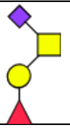	821.3041	821.3045	17.5	*43.4*	1	1	1	1	0	0
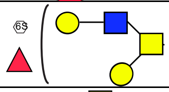	975.2982	975.2981	17.8	*10.7*	2	2	1	0	0	1
	975.2972		18.5	*0.12*						
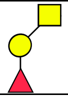	530.2082	530.2090	15.7	*10.8*	1	1	1	0	0	0
	530.2102		14.4	*0.06*						
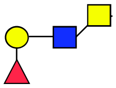	733.2874	733.2884	18.8	*9.10*	1	2	1	0	0	0
	733.2858		17.1	*0.04*						
	733.2865		20.0	*0.03*						
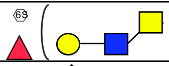	813.2447	813.2452	17.4	*4.79*	1	2	1	0	0	1
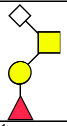	837.2990	837.2994	18.2	*3.89*	1	1	1	0	1	0
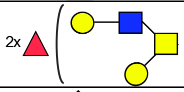	1041.3995	1041.3992	21.0	*3.39*	2	2	2	0	0	0
	1041.3983		21.4	*0.27*						
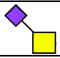	513.1927	513.1937	15.9	*3.21*	0	1	0	1	0	0
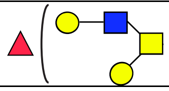	895.3405	895.3412	20.4	*2.08*	2	2	1	0	0	0
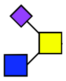	716.2722	716.2731	17.3	*0.94*	0	2	0	1	0	0
	716.2717		17.0	*0.86*						
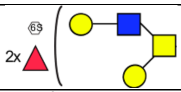	1121.3557	1121.3560	18.7	*0.93*	2	2	2	0	0	1
	1121.3558		19.0	*0.13*						
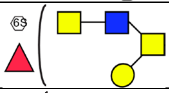	1016.3242	1016.3246	17.7	*0.87*	1	3	1	0	0	1
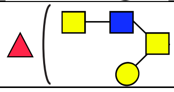	936.3664	936.3678	20.9	*0.58*	1	3	1	0	0	0
	936.3658		20.5	*0.07*						
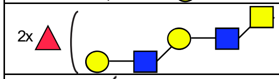	1244.4779	1244.4785	21.7	*0.54*	2	3	2	0	0	0
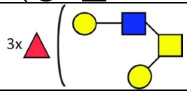	1187.4576	1187.4571	22.1	*0.40*	2	2	3	0	0	0

O-glycan structures identified in BSM after beta elimination and analysis at pH 7.8.

Composition	Observed m/z [M-H]⁻	Calculated m/z [M-H]⁻	Retention time (min)	Relative abundancy (%)	Hex	HexNAc	Fuc	Neu5Ac	Neu5Gc	Sulf
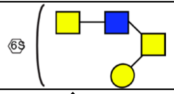	870.2661	870.2667	17.7	*0.36*	1	3	0	0	0	1
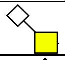	529.1873	529.1886	16.8	*0.35*	0	1	0	0	1	0
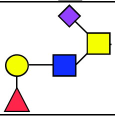	1024.3822	1024.3838	18.7	*0.34*	1	2	1	1	0	0
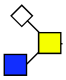	732.2665	732.2680	17.7	*0.16*	0	2	0	0	1	0
	732.2658		18.0	*0.14*						
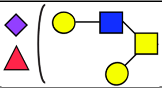	1186.4371	1186.4367	18.9	*0.23*	2	2	1	1	0	0
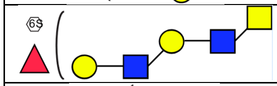	1178.3766	1178.3774	19.1	*0.21*	2	3	1	0	0	1
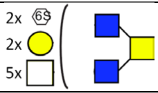	1063.3317	1063.3330	23.6	*0.19*	2	8	0	0	0	2
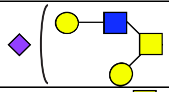	1040.3777	1040.3788	19.4	*0.18*	2	2	0	1	0	0
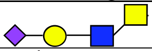	878.3248	878.3259	18.5	*0.12*	1	2	0	1	0	0
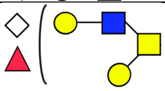	1202.4309	1202.4316	19.5	*0.10*	2	2	1	0	1	0
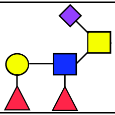	1170.4400	1170.4417	19.6	*0.09*	1	2	2	1	0	0
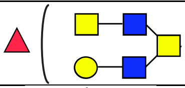	1139.4469	1139.4472	22.4	*0.06*	1	4	1	0	0	0
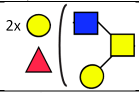	1057.3931	1057.3941	21.8	*0.06*	3	2	1	0	0	0
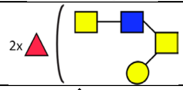	1082.4236	1082.4257	22.1	*0.04*	1	3	2	0	0	0
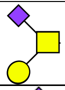	675.2445	675.2466	17.6	*0.04*	1	1	0	1	0	0
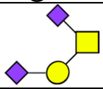	966.3404	966.3420	17.9	*0.04*	1	1	0	2	0	0
